# Placebo vs Amoxicillin for Nonsevere Fast-Breathing Pneumonia in Malawian Children Aged 2 to 59 Months: A Double-blind, Randomized Clinical Noninferiority Trial

**DOI:** 10.1001/jamapediatrics.2018.3407

**Published:** 2018-11-12

**Authors:** Amy Sarah Ginsburg, Tisungane Mvalo, Evangelyn Nkwopara, Eric D. McCollum, Chifundo B. Ndamala, Robert Schmicker, Ajib Phiri, Norman Lufesi, Rasa Izadnegahdar, Susanne May

**Affiliations:** 1 Save the Children Federation, Inc, Fairfield, Connecticut; 2 University of North Carolina Project, Lilongwe Medical Relief Fund Trust, Tidziwe Centre, Lilongwe, Malawi; 3 Eudowood Division of Pediatric Respiratory Sciences, Department of Pediatrics, Johns Hopkins School of Medicine, Baltimore, Maryland; 4 Department of International Health, Johns Hopkins Bloomberg School of Public Health, Baltimore, Maryland; 5 Department of Biostatistics, University of Washington Clinical Trial Center, Seattle; 6 Department of Pediatrics and Child Health, College of Medicine, University of Malawi, Chichiri, Blantyre; 7 Acute Respiratory Infection and Emergency Triage Assessment and Treatment, Malawi Ministry of Health, Lilongwe; 8 Bill & Melinda Gates Foundation, Seattle, Washington

## Abstract

**Importance:**

Pneumonia is the leading infectious killer of children. Rigorous evidence supporting antibiotic treatment of children with nonsevere fast-breathing pneumonia in low-resource African settings is lacking.

**Objective:**

To assess whether treatment with placebo for nonsevere fast-breathing pneumonia is substantively less effective than 3 days of treatment with amoxicillin.

**Design, Setting, and Participants:**

This double-blind, 2-arm, randomized clinical noninferiority trial with follow-up of 14 days screened 1343 HIV-uninfected children aged 2 to 59 months with nonsevere fast-breathing pneumonia at outpatient departments of hospitals in Lilongwe, Malawi, Africa, between June 2016 and June 2017.

**Interventions:**

Placebo or amoxicillin dispersible tablets administered twice daily for 3 days.

**Main Outcomes and Measures:**

The primary end point was the proportion of children failing treatment by day 4 with a relative noninferiority margin of 1.5 times the failure rate in the amoxicillin group. Primary analyses were performed based on the intention-to-treat principle. Planned secondary analyses included treatment failure or relapse by day 14.

**Results:**

In total, 1126 children were randomized to 3 days of amoxicillin (n = 564) or placebo (n = 562) therapy. Baseline demographic and clinical characteristics were similar between the groups. For the entire study population, the mean (SD) age was 21.3 (15.1) months, and 601 (53.4%) were female. After an interim analysis, the data safety monitoring board stopped the study because children receiving amoxicillin had a 4.0% (22 of 552 with outcome data) treatment failure rate by day 4, whereas children receiving placebo had a 7.0% (38 of 543) treatment failure rate (adjusted relative risk, 1.78; 95% CI, 1.07%-2.97%; adjusted absolute difference, 3.0%; 95% CI, 0.4%-5.7%). Among children with known day 14 outcomes, 56 of 552 (10.1%) receiving amoxicillin and 64 of 543 (11.8%) receiving placebo had either treatment failure by day 4 or relapse by day 14 (relative risk, 1.16; 95% CI, 0.83%-1.63%; absolute difference, 1.6%; 95% CI, −2.1% to 5.4%). There were no deaths.

**Conclusions and Relevance:**

In HIV-uninfected children aged 2 to 59 months in a malaria-endemic region of Malawi, placebo treatment of nonsevere fast-breathing pneumonia was significantly inferior to treatment with amoxicillin. However, by day 4, approximately 93% of children receiving placebo were without treatment failure, and there was no significant difference between groups in treatment failure or relapse by day 14. The number of children with nonsevere fast-breathing pneumonia that needed amoxicillin treatment for 1 child to benefit was 33.

**Trial Registration:**

ClinicalTrials.gov Identifier: NCT02760420

## Key Points

**Question:**

Are antibiotics necessary for the treatment of nonsevere fast-breathing pneumonia in children?

**Findings:**

In this double-blind, randomized clinical noninferiority trial that included 1126 HIV-uninfected children aged 2 to 59 months in a malaria-endemic region of Malawi, Africa, placebo treatment of nonsevere fast-breathing pneumonia was significantly inferior to 3 days of amoxicillin treatment with respect to treatment failure at day 4. Fast-breathing pneumonia resolved by day 4 in 93% of children without the use of the antibiotic.

**Meaning:**

Without amoxicillin treatment, 7% of Malawian children with nonsevere fast-breathing pneumonia failed treatment by day 4, and treating 33 children with amoxicillin was necessary for 1 child to benefit.

## Teaser

This double-blind, randomized clinical noninferiority trial evaluates whether placebo treatment is significantly inferior to 3 days of oral amoxicillin treatment in HIV-uninfected children aged 2 to 59 months with nonsevere fast-breathing pneumonia in a malaria-endemic region of Malawi, Africa.

## Introduction

Approximately 920 000 children die of pneumonia annually before their fifth birthday.^1^ There is a critical need to provide greater access to appropriate and effective treatment. Treatment of bacterial pneumonia requires an effective antibiotic used in adequate doses for an appropriate duration. A 3-day course of twice-daily amoxicillin is recommended by the World Health Organization (WHO) as first-line treatment of nonsevere fast-breathing pneumonia among immune-competent children aged 5 years or less.^2^ The WHO’s integrated management of childhood illness (IMCI) guidelines identify fast-breathing pneumonia in children with cough or difficult breathing who demonstrate fast breathing without WHO danger signs.^3^ However, not all cases of fast breathing are bacterial pneumonia. Indeed, the majority of fast-breathing pneumonia cases are likely not pneumonia at all. There are many reasons that a child might demonstrate fast breathing, and not all indicate disease.^4,5,6,7^ Thus, isolated fast breathing may not be appropriately sensitive nor specific for diagnosing pneumonia,^8^ making antibiotic treatment unnecessary. Although it is relatively well tolerated among children, amoxicillin has side effects and causes serious but rare adverse events.^9^ Furthermore, inappropriate antibiotic use may lead to antibiotic resistance.^10,11,12,13,14,15^

Current case management guidelines maximize diagnostic sensitivity over specificity, potentially resulting in antibiotic overuse.^16^ Existing research suggests that most fast-breathing pneumonia cases will resolve without antibiotic therapy. In a study evaluating fever etiology among outpatient children in Tanzania, the authors found that, in the absence of critical illness and malaria, most febrile children could be treated supportively without antibiotics.^17^ There is a need to appropriately treat children with bacterial pneumonia with antibiotics and to not unnecessarily treat children with illnesses unresponsive to antibiotics.

Studies in Asia have evaluated the effectiveness of 3-day amoxicillin treatment of fast-breathing pneumonia;^2,6,18,19^ however, evidence is needed to evaluate whether any antibiotic treatment is required.^20^ There have been multiple calls for carefully designed, scientifically sound studies repeated in different geographic regions to assess the need for antibiotic treatment of fast-breathing pneumonia. Given the paucity of such data from Africa, there is a critical need for African-specific research in malaria-endemic settings to establish optimal management of fast-breathing pneumonia in children.

## Methods

### Study Design

The primary objective of this prospective, double-blind, 2-arm, randomized controlled noninferiority trial was to investigate whether treatment with placebo in HIV-uninfected children aged 2 to 59 months with nonsevere fast-breathing pneumonia in a malaria-endemic region of Malawi was (null hypothesis) substantively less effective than 3 days of treatment with amoxicillin. An innovative noninferiority design was developed based on the assertion that placebo could not be expected to be more beneficial than amoxicillin with respect to the primary outcome of treatment failure (TF) by day 4 but could be expected to be only slightly worse than amoxicillin (alternative hypothesis). Children aged 2 to 59 months meeting the fast-breathing pneumonia case definition (eAppendix 1 in Supplement 1) at the outpatient departments of Kamuzu Central Hospital (KCH) and Bwaila District Hospital in Lilongwe, Malawi, Africa, were screened by 2 of us (T.M. and C.B.N.) to determine eligibility (eAppendix 1 in Supplement 1). The study was conducted in accordance with the International Conference on Harmonisation, Good Clinical Practice, and the Declaration of Helsinki 2008^21^ and was approved by the Western Institutional Review Board of Washington in the United States, the College of Medicine Research and Ethics Committee in Blantyre, Malawi, and the Malawi Pharmacy, Medicines and Poisons Board. All children’s parents or legal guardians provided written informed consent to participate in this trial. The trial protocol is provided in Supplement 2.

### Procedures

On day 1 after enrollment, eligible children were randomized in a 1:1 ratio to receive 3 days of either placebo dispersible tablets (intervention) or amoxicillin dispersible tablets, 250 mg, (control) in 2 divided doses based on age ranges (500 mg/d for children 2-11 months of age, 1000 mg/d for children 12-35 months, and 1500 mg/d for children 36-59 months), the current WHO-recommended therapy for HIV-uninfected children.^2^ Study drugs were identical in appearance, smell, taste, dispersion activity, and packaging. Randomization was stratified by age groups (2-11, 12-35, and 36-59 months) using blocks of size 2, 4, and 6. Other than the unblinded biostatisticians, pharmacists, monitors, and data safety monitoring board members, everyone on the study team was blinded to each child’s assigned treatment group.

Enrollment was initially conducted solely at KCH (phase 1) and then transitioned on September 20, 2016, to Bwaila District Hospital (phase 2) after KCH introduced bypass fees, which reduced patient volumes. Bwaila District Hospital enrollees were transferred to KCH for additional evaluation, observation, and admission, if necessary. To maximize safety, all enrollees were observed for at least 2 to 8 hours before discharge. Children with no fever and a respiratory rate (RR) below the enrollment threshold were discharged after 2 hours; other children remained under observation. Children aged less than 6 months, children with moderate malnutrition (11.5-13.5 cm mid–upper arm circumference), or febrile children with a negative malaria rapid diagnostic test result were hospitalized overnight and assessed for discharge on day 2.

During observation, if a child’s condition deteriorated (new WHO IMCI danger sign, hypoxemia, RR 10 breaths higher than that at enrollment, or severe respiratory distress), the child was hospitalized and counted as a TF (eAppendix 1 in Supplement 1). During discharge, an oxygen saturation less than 93% or very fast breathing required continued hospital monitoring but was not considered a TF. If discharge criteria were not met by day 3, the child’s status was considered a TF.

Caregivers received phone calls on days 1 to 3 and were evaluated in the clinic or home on days 2 to 4 and day 14. During follow-up, all children were assessed for TF or relapse (eAppendix 1 in Supplement 1) and study drug adherence at all scheduled and unscheduled visits. Most children with TF or relapse were hospitalized and treated with second-line intravenous antibiotics. Once second-line antibiotics were started, the child was considered nonadherent to the randomized treatment.

### Outcomes

The primary end point was the proportion of children with day 4 TF (eAppendix 1 in Supplement 1). Secondary end points were proportions of children with relapse (days 5-14 among children without TF before or on day 4), with day 4 TF or relapse by day 14, with TF among those with baseline malaria, and by age group. All adverse events were assessed and managed per KCH standard clinical practice, documented, followed up, and treated until resolution or stabilization. All serious adverse events (eAppendix 1 in Supplement 1) were reported for review within 24 hours.

### Statistical Analysis

The statistical analysis plan is provided in eAppendix 2 in Supplement 1. A relative noninferiority margin of 1.5 times the TF rate in the amoxicillin group was chosen based on an anticipated TF rate in the amoxicillin group of 4% to 10%. This noninferiority margin was selected after extensive discussions among the investigators (including A.S.G. and S.M.) regarding the TF rate for placebo that would be acceptable to clinicians, considering the anticipated potential TF rate in the amoxicillin group and potential for enrollment in the study. Adjusting for 2 formal interim analyses (the O’Brien-Fleming boundary for early noninferiority^22^ and the Pocock boundary for early inferiority^23^), enrolling 2000 children (1000 per group) provided 84.2% power when the TF rate was 7% in the amoxicillin group and 64.8% power when the TF rate was 4% in the amoxicillin group, assuming equal TF rates in both groups. These power calculations took into account a dropout rate of 5% and assumed a 1-sided α of .025 for a test of a difference in proportions. The test was initially conceptualized as 1-sided because of the assertion that placebo could not be more beneficial than amoxicillin regarding TF; however, 2-sided 95% CIs are also presented because placebo was inferior to amoxicillin (the harmful direction). Primary analyses were performed based on the intention-to-treat principle using linear regression adjusted for age groups, study phase, and sex and using robust standard errors based on the Huber-White sandwich estimator.^24,25^ Given that its use would be justified because the sample size was sufficiently large, linear regression analysis was conducted for this binary outcome to model differences in rates.^26^ Relative risks are also presented. Sensitivity analyses were performed using multiple imputations^27^ and tipping point analyses. Analyses of secondary end points used robust standard errors unadjusted for interim analyses or any other factor. Further analysis details are provided in eAppendix 6 in Supplement 1. Statistical analyses were conducted using R, versions 3.2.5 and 2.14.1 (the R Foundation).

## Results

Enrollment started June 7, 2016, with formal interim analysis conducted after one-third of the children were enrolled. In the interim analysis, the early inferiority boundary was crossed, and after review of additional data, the data safety monitoring board members recommended pausing enrollment and analyzing data from the children already enrolled at the time of the review. After subsequent review, the board recommended stopping the study. The last visit was completed June 20, 2017.

In total, 1343 children were screened, of which 61 were eligible but refused enrollment consent and 166 were ineligible (eAppendix 3 in Supplement 1); however, 10 of these 166 were enrolled (Figure). Thus, a total of 1126 children were enrolled, with 564 receiving amoxicillin and 562 receiving placebo. The primary outcome was available for 552 children (97.9%) in the amoxicillin group and 543 children (96.6%) in the placebo group. Baseline demographic and clinical characteristics were similar between the groups (Table 1). There were no substantive differences between the treatment groups in the distribution of RR by age groups.

**Figure.**
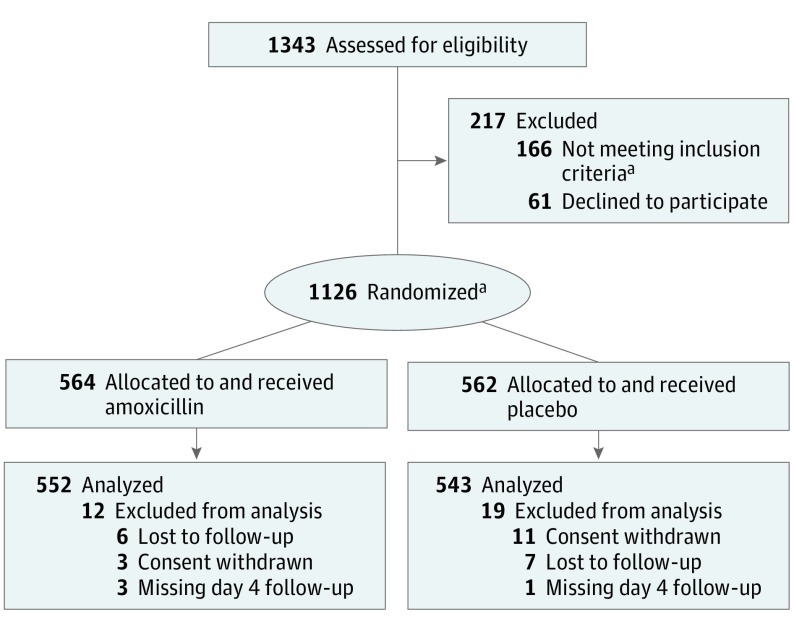
CONSORT Diagram by Treatment Group ^a^Of the 166 ineligible children, 10 were enrolled. CONSORT indicates Consolidated Standards of Reporting Trials.

**Table 1.** Child Demographic and Clinical Characteristics at Enrollment by Treatment Group

Characteristic	Children, No. (%)		
	Amoxicillin (n = 564)	Placebo (n = 562)	Overall (n = 1126)
Age, mo			
2-11	196 (34.8)	196 (34.9)	392 (34.8)
12-35	255 (45.2)	254 (45.2)	509 (45.2)
36-59	113 (20.0)	112 (19.9)	225 (20.0)
Sex			
Male	271 (48.0)	254 (45.2)	525 (46.6)
Female	293 (52.0)	308 (54.8)	601 (53.4)
Height/weight *z* score^a^			
<−3	0	1 (0.2)	1 (0.1)
−2 to −3	5 (0.9)	9 (1.6)	14 (1.2)
>−2	558 (98.9)	552 (98.2)	1110 (98.6)
Mid–upper arm circumference, cm^a^			
<11.5	0	0	0
11.5-13.5	32 (5.7)	45 (8.0)	77 (6.8)
>13.5	531 (94.1)	517 (92.0)	1048 (93.1)
Respiratory rate, mean (SD), breaths/min^b^			
Age 2-11 mo			
<50	1 (0.2)	0	1 (0.1)
50-59	133 (23.6)	146 (26.0)	279 (24.8)
≥60	62 (11.0)	50 (8.9)	112 (9.9)
Age 12-59 mo			
<40	0	0	0
40-49	234 (41.5)	233 (41.5)	467 (41.5)
≥50	134 (23.8)	133 (23.7)	267 (23.7)
Oxygen saturation, %^b^			
<90	0	0	0
90-92	0	0	0
≥93	564 (100)	562 (100)	1126 (100)
Axillary temperature, °C^b^			
<38	386 (68.4)	405 (72.1)	791 (70.2)
≥38	178 (31.6)	157 (27.9)	335 (29.8)
Heart rate, mean (SD), beats/min^a^	142.3 (18.1)	142.4 (17.2)	142.4 (17.6)
Pneumococcal vaccine			
Received 3 doses	298 (52.8)	321 (57.1)	619 (55.0)
Received <3 doses or unknown	266 (47.2)	241 (42.9)	507 (45.0)
Pentavalent vaccine			
Received 3 doses	301 (53.4)	319 (56.8)	620 (55.1)
Received <3 doses or unknown	263 (46.6)	243 (43.2)	506 (44.9)
Caregiver assessment at enrollment			
Fever^a^			
Yes	476 (84.4)	469 (83.5)	945 (83.9)
No. of days, mean (SD)	2.7 (1.1)	2.8 (1.4)	2.7 (1.2)
Cough^a^			
Yes	546 (96.8)	547 (97.3)	1093 (97.1)
No. of days, mean (SD)	2.9 (1.2)	3.1 (1.5)	3.0 (1.4)
Difficult breathing^a^			
Yes	180 (31.9)	180 (32.0)	360 (32.0)
No. of days, mean (SD)	2.5 (0.9)	2.6 (1.3)	2.5 (1.1)

^a^ Data not available for all randomized children.

^b^ Larger value between screening and enrollment visits.

By day 4, amoxicillin recipients had a TF rate of 4.0% (22 of 552 with day 4 outcome), and placebo recipients had a TF rate of 7.0% (38 of 543), resulting in an adjusted TF relative risk of 1.78 (95% CI, 1.07%-2.97%) and an absolute TF rate difference (intention-to-treat primary analysis) of 3.0% (95% CI, 0.4%-5.7%) (Table 2). Among the children without TF by day 4, 34 of 530 children (6.4%) had relapse by day 14 in the amoxicillin group compared with 26 of 505 children (5.1%) in the placebo group, representing a relative risk of 0.80 (95% CI, 0.49%-1.32%). When considering both TF before or by day 4 and relapse by day 14, 56 of 552 children (10.1%) in the amoxicillin group and 64 of 543 children (11.8%) in the placebo group met criteria (relative risk, 1.16; 95% CI, 0.83%-1.63%). Additional secondary outcome results are provided in Table 2. The higher TF rate in the placebo group was consistent across prespecified subgroups defined by age group, RR, malnutrition, malaria, or by whether a child was known to have received 3 doses of pneumococcal conjugate vaccine or pentavalent vaccine or not (Table 2; eAppendix 4 in Supplement 1). The amount of missing primary outcome data was low (overall, 31 data points [2.8%]: 12 data points in the amoxicillin group and 19 in the placebo group). Estimates derived from multiple imputations for missing outcome data were similar to the results from the complete case analysis. The results of a tipping point analysis indicated that TF rates among those with missing data would need to be in the opposite direction of that observed (ie, higher TF rates in the amoxicillin than in the placebo groups) for the complete data to change the differences in the TF rate from significant to nonsignificant.

**Table 2.** Outcomes by Treatment Group

Outcome	Children, No./Total No. (%)		Relative Risk (95% CI)	Difference, % (95% CI)
	Amoxicillin (n = 564)	Placebo (n = 562)		
Primary				
Treatment failure on or prior to day 4^a^	22/552 (4.0)	38/543 (7.0)	1.78 (1.07 to 2.97)	3.0 (0.4 to 5.7)
Secondary a priori				
Relapse on or prior to day 14 if cured by day 4^b^	34/530 (6.4)	26/505 (5.1)	0.80 (0.49 to 1.32)	1.3 (−4.1 to 1.6)
Treatment failure or relapse on or prior to day 14	56/552 (10.1)	64/543 (11.8)	1.16 (0.83 to 1.63)	1.6 (−2.1 to 5.4)
As treated referent ≥80 amoxicillin doses^c^	14/541 (2.6)			
Partial adherence to amoxicillin (0 < adherence < 80)	3/6 (50.0)		19.3 (7.45 to 50.1)	47.0 (6.8 to 87.1)
Adherent to placebo^d^		36/541 (6.7)	2.57 (1.40 to 4.71)	4.1 (1.6 to 6.6)
Multiple imputation^e^			1.76 (1.06 to 2.94)	3.2 (0.4 to 5.9)
Subgroups a priori				
Age groups, mo				
2-11	10/189 (5.3)	15/187 (8.0)	1.52 (0.70 to 3.29)	2.7 (−2.3 to 7.8)
12-35	9/252 (3.6)	17/245 (6.9)	1.87 (0.75 to 4.69)	3.4 (−0.6 to 7.3)
36-59	3/111 (2.7)	6/111 (5.4)	2.21 (0.79 to 6.16)	2.7 (−2.5 to 7.9)
Fast breathing, RR^f^				
2-11 mo				
50-59	5/131 (3.8)	12/140 (8.6)	2.25 (0.81 to 6.20)	4.8 (−0.9 to 10.4)
60-69	4/52 (7.7)	3/46 (6.5)	0.85	−1.2
≥70	1/5 (20.0)	0/1 (0)	0	−20.0
12-23 mo				
40-49	3/78 (3.8)	4/73 (5.5)	1.42	1.6
50-59	2/64 (3.1)	6/49 (12.2)	3.92	9.1
≥60	2/13 (15.4)	1/8 (12.5)	0.81	−2.9
24-59 mo				
40-49	4/153 (2.6)	8/155 (5.2)	1.97 (0.61 to 6.42)	2.5 (−1.8 to 6.9)
50-59	1/50 (2.0)	3/66 (4.5)	2.27	2.5
≥60	0/5 (0)	1/5 (20.0)	ND	20.0
Mid–upper arm circumference, cm^f^				
<11.5	0	0	ND	ND
11.5-13.5	1/32 (3.1)	6/43 (14.0)	4.46	10.8
>13.5	21/519 (4.0)	32/500 (6.4)	1.58 (0.92 to 2.71)	2.4 (−0.4 to 5.1)
Malaria				
Positive	0/72 (0)	1/65 (1.5)	ND	1.5
Negative	22/480 (4.6)	37/478 (7.7)	1.69 (1.01 to 2.82)	3.2 (0.1 to 6.2)

Abbreviations: ND, not determined; RR, respiratory rate.

^a^ Difference and 95% CI adjusted for stratification variables age and phase.

^b^ Of those without treatment failure on or prior to day 4.

^c^ Excluding treatment failures within 2 hours; difference and CI adjusted for age group, sex, and study phase.

^d^ All children in the placebo group were considered adherent to placebo in the as-treated analysis.

^e^ Covariates used in imputation were treatment group, age group, sex, and mother’s educational level.

^f^ Data not available for all randomized children.

The percentage of children with at least 1 serious adverse event between enrollment and day 14 was higher in the placebo group (54 of 562, 9.6%) than in the amoxicillin group (44 of 564, 7.8%) (Table 3). This outcome was primarily caused by a higher proportion of children with pneumonia-related serious adverse events in the placebo group. There were no deaths. Caregiver-reported adherence was high, with 517 of 564 (91.7%) reporting adherence with all doses in the amoxicillin group and 509 of 562 (90.6%) reporting adherence with all doses in the placebo group (eAppendix 5 in Supplement 1).

**Table 3.** Serious Adverse Events by Treatment Group

**Serious Adverse Event**^a^	Children, No. (%)		
	Amoxicillin (n = 564)	Placebo (n = 562)	Overall (n = 1126)
Children with at least 1 serious adverse event^b^	44 (7.8)	54 (9.6)	98 (8.7)
Pneumonia^c^	34 (6.0)	42 (7.5)	76 (6.7)
Fast-breathing pneumonia^d^	7 (1.2)	12 (2.1)	19 (1.7)
Chest-indrawing pneumonia	17 (3.0)	18 (3.2)	35 (3.1)
Danger sign pneumonia	5 (0.9)	9 (1.6)	14 (1.2)
Chest radiograph–confirmed pneumonia^e^	4 (0.7)	4 (0.7)	8 (0.7)
Pneumonia^f^	1 (0.2)	0	1 (0.1)
Nonrespiratory	12 (2.1)	13 (2.3)	25 (2.2)
Acute gastroenteritis	4 (0.7)	4 (0.7)	8 (0.7)
Fever	1 (0.2)	4 (0.7)	5 (0.4)
Malaria	2 (0.4)	1 (0.2)	3 (0.3)
Convulsion	1 (0.2)	1 (0.2)	2 (0.2)
Urinary tract infection	0	2 (0.4)	2 (0.2)
Vomiting	2 (0.4)	0	2 (0.2)
Anemia	0	1 (0.2)	1 (0.1)
Epistaxis	1 (0.2)	0	1 (0.1)
Febrile convulsion	1 (0.2)	0	1 (0.1)

^a^ Children may have more than 1 serious adverse event.

^b^ Occurring any time after study drug is administered to child up to 14 days after enrollment.

^c^ One child in the placebo group had a danger sign pneumonia serious adverse event before day 4 and a fast-breathing pneumonia serious adverse event after day 4.

^d^ Of the 19 fast-breathing pneumonia serious adverse events, 4 were treatment failures (in the placebo group) on day 4 and met additional treatment failure criteria. The remaining 15 serious adverse events were recurrences of fast-breathing pneumonia and, therefore, relapses after day 4.

^e^ Chest radiograph–confirmed pneumonia serious adverse events did not demonstrate fast breathing, chest indrawing, or any danger signs; however, pneumonia was diagnosed through positive results on chest radiographs.

^f^ For 1 child, no type of pneumonia was specified.

## Discussion

We evaluated 3 days of oral placebo administration vs oral amoxicillin treatment among 1126 HIV-uninfected children aged 2 to 59 months presenting with WHO–defined nonsevere fast-breathing pneumonia in a malaria-endemic region of Malawi, Africa. Our results showed that, compared with children who received placebo, children who received amoxicillin had a significantly lower TF rate on or before day 4. The direction of the estimated effect was the same across prespecified subgroups and in an as-treated analysis. In a post hoc analysis examining different RRs, the direction of difference in the TF rate was consistent in children presenting with the lowest fast-breathing RR and regardless of number of pneumococcal conjugate or pentavalent vaccine doses. However, by day 4, approximately 93% of children were without TF in the placebo group. Although there was no significant difference between the groups at 14 days, our study could not rule out meaningful differences in TF or relapse by day 14, which were estimated to be less than 12% in both groups. The number of nonsevere fast-breathing pneumonia cases needed to treat with amoxicillin for 1 child to benefit was 33. It might be argued that such a large number needed to treat to benefit 1 child is acceptable relative to the low risks associated with taking antibiotics. However, it might also be argued that efforts should focus on identifying and providing antibiotics to those children who truly need them to prevent TF or relapse or focus on identifying and providing only supportive care to those children who recover without antibiotics.

Although placebo was inferior to amoxicillin for treatment of nonsevere fast-breathing pneumonia, the vast majority of children in the placebo group recovered without amoxicillin. As in previous studies,^7^ this observation suggests that true bacterial pneumonia among children with fast breathing in the present study appeared to have been low, implying that fast breathing might be neither an appropriately sensitive nor specific sign of bacterial pneumonia. It also suggests that in a small subgroup of children receiving a diagnosis of WHO-defined nonsevere fast-breathing pneumonia, antibiotics appeared to reduce the absolute rate of TF by a 3.0% adjusted difference (relative risk of TF, 1.78 for the comparison of children who received placebo vs those who received amoxicillin). Thus, in a small subset of fast-breathing children, amoxicillin seemed to be of benefit, but it was unclear whether the benefit was derived from the appropriate treatment of bacterial pneumonia or the treatment of some other infection or non-antibacterial inflammatory process.

The results of the present study suggest that it may be advisable to schedule a follow-up visit on day 4 for reevaluation and treatment if necessary for low-risk children who are available for follow-up. For those whose follow-up cannot be assured—and this may be the majority of children in routine settings in sub-Saharan Africa—treatment with amoxicillin may be warranted; it is unclear whether those children would require a longer course of amoxicillin. More research is needed regarding the duration of the amoxicillin treatment given the 6% relapse vs the 4% TF rates among children with nonsevere fast-breathing pneumonia who received 3 days of amoxicillin.

The WHO recommendations to use antibiotics to treat fast breathing in a child with cough or difficulty breathing are based on low-quality evidence.^28^ A 2016 Cochrane review assessing antibiotic treatment of undifferentiated acute respiratory infection in children less than 5 years of age found that there is insufficient evidence for antibiotic use as a means of preventing suppurative complications, such as pneumonia.^29^ In the United States, clinical practice guidelines established by the Pediatric Infectious Diseases Society and the Infectious Diseases Society of America note that, for outpatients, “antimicrobial therapy is not routinely required for preschool-aged children with community-acquired pneumonia, because viral pathogens are responsible for the great majority of clinical disease (strong recommendation; high-quality evidence).”^30^^(pe30)^ While *Haemophilus influenzae* type b (Hib) and pneumococcal conjugate vaccines are being scaled up—the Hib conjugate vaccine was introduced in Malawi in 2002 as part of the Expanded Programme for Immunization, and the pneumococcal conjugate vaccine was added to the national strategy in 2011—the etiology and epidemiology of community-acquired pneumonia is changing. For example, a recent pneumococcal conjugate vaccine effectiveness study in Malawi found a reduction in the severest forms of clinical and hypoxemic pneumonia alongside a seemingly paradoxical increase in fast-breathing pneumonia cases. The authors speculated that this likely represented a vaccine-induced shift away from more severe combined viral and pneumococcal disease to less severe pneumococcal-free viral disease that still met the fast-breathing pneumonia definition.^31^ In 2015, the Expanded Programme for Immunization in Malawi reported 88% coverage for both Hib and pneumococcal conjugate vaccines.

### Limitations

The limitations to our study included strict inclusion and exclusion criteria, the absence of laboratory or radiology testing, and the close monitoring and follow-up, which limits the generalizability of our results to routine programmatic care settings. Pneumonia is frequently considered a single entity rather than a clinical syndrome encompassing several underlying factors. This makes interpretation of the results challenging. Without etiologic information, we could only note the effect of the intervention on the clinical syndrome of pneumonia.

Follow-up care and monitoring of enrolled children generally exceeded local standards of care; thus, the TF rate may have been influenced by both the high quality of care provided and by the high awareness and vigilance for identifying TF. It may be that those children identified as failing treatment would have recovered without antibiotics had we taken a watchful waiting approach and not intervened with antibiotic treatment. However, opportunities for follow-up and access to care are often issues in low-resource settings. In addition, treatment approaches vary widely among countries and regions. The routine pediatric HIV testing included in this study protocol, although recommended, is not rigorously implemented during routine care in low-resource, HIV-endemic settings.^32^ In areas where HIV endemicity is high or where severe acute malnutrition or other predisposing conditions for bacterial disease is common, it may be reasonable to expect a higher TF rate among those not treated with antibiotics. As such, our results might not be generalizable across different regions, settings, or nontrial conditions. Specifically, the percentages of TF and relapse rates observed in the present study might underestimate true TF and relapse rates.

## Conclusions

In our population of HIV-uninfected children with nonsevere fast-breathing pneumonia in Malawi, Africa, placebo administration was inferior to 3 days of amoxicillin treatment. However, 93% of children with WHO-defined fast-breathing pneumonia and without antibiotic treatment were without TF by day 4. There were no deaths. In addition, the number needed to treat was 33, which is considered high. Further research is needed to better define which children would truly benefit from antibiotic therapy.

## Notes

Supplement 1. **eAppendix 1.** Study Definitions **eAppendix 2.** Statistical Analysis Plan **eAppendix 3.** Reasons for Study Ineligibility **eAppendix 4.** Posteriori Analysis by Treatment Group **eAppendix 5.** Treatment Adherence by Age and Treatment Group **eAppendix 6.** Supplementary Material

Supplement 2. **Trial Protocol**
