# Supplementary material for: Placebo vs Amoxicillin for Nonsevere Fast-Breathing Pneumonia in Malawian Children Aged 2 to 59 Months: A Double-blind, Randomized Clinical Noninferiority Trial
Source: JAMA Pediatr. 2018 Nov 12;173(1):21–8. doi: 10.1001/jamapediatrics.2018.3407 (PMC6583426; doi:10.1001/jamapediatrics.2018.3407)
Supplement: Supplement 1. — eAppendix 1. Study Definitions eAppendix 2. Statistical Analysis Plan eAppendix 3. Reasons for Study Ineligibility eAppendix 4. Posteriori Analysis by Treatment Group eAppendix 5. Treatment Adherence by Age and Treatment Group eAppendix 6. Supplementary Material [file jamapediatr-173-21-s001.pdf]

Supplementary Online Content

Ginsburg AS, Mvalo T, Nkwopara E, et al. Placebo vs amoxicillin for nonsevere fast-breathing pneumonia in Malawian children aged 2 to 59 months: a double-blind, randomized clinical noninferiority trial. *JAMA Pediatr.* Published online November 12, 2018. doi:10.1001/jamapediatrics.2018.3407

eAppendix 1. Study Definitions

eAppendix 2. Statistical Analysis Plan

eAppendix 3. Reasons for Study Ineligibility

eAppendix 4. Posteriori Analysis by Treatment Group

eAppendix 5. Treatment Adherence by Age and Treatment Group

eAppendix 6. Supplementary Material

This supplementary material has been provided by the authors to give readers additional information about their work.

© 2018 Ginsburg AS et al. *JAMA Pediatrics.*

eAppendix 1. Study definitions

**Study definitions**	
Non-severe fast-breathing pneumonia	Cough < 14 days or difficulty breathing AND fast-breathing for age
Fast-breathing for age	Respiratory rate ≥50 breaths per minute (for children 2 to <12 months of age) or ≥40 breaths per minute (for children ≥12 months of age)
Very fast-breathing for age	≥70 breaths per minute (for children 2 to <12 months of age) or ≥60 breaths per minute (for children ≥12 months of age).
Severe respiratory distress	Grunting, nasal flaring, head nodding, and/or chest-indrawing
Hypoxemia	Arterial oxyhemoglobin saturation (SpO_2) < 90% in room air, as assessed non-invasively by a pulse oximeter
World Health Organization (WHO) Integrated Management of Childhood Illness (IMCI) general danger signs	Lethargy or unconsciousness, convulsions, vomiting everything, inability to drink or breastfeed
Severe acute malnutrition	Weight for height/length < -3 SD, mid-upper arm circumference (MUAC) <11·5 cm, or peripheral edema
Severe malaria	Positive malaria rapid diagnostic test with any WHO IMCI general danger sign, stiff neck, abnormal bleeding, clinical jaundice, or hemoglobinuria
HIV-1 exposure	Children <24 months of age with a HIV-infected mother
Serious adverse event	Adverse event that:<br>• Results in death<br>• Is life threatening<br>• Requires inpatient hospitalization or prolongation of existing hospitalization<br>• Results in persistent or significant disability/incapacity<br>• Is a medical event, based on appropriate medical judgment, that may jeopardize the health of the participating child or require medical or surgical intervention to prevent 1 of the outcomes listed
**Eligibility criteria**	
Inclusion criteria	• 2-59 months of age<br>• Cough <14 days or difficulty breathing<br>• Fast-breathing for age

© 2018 Ginsburg AS et al. *JAMA Pediatrics.*

Exclusion criteria	• Severe respiratory distress<br>• Hypoxemia<br>• Resolution of fast-breathing after bronchodilator challenge, if wheezing at screening examination<br>• WHO IMCI general danger signs<br>• Stridor when calm<br>• HIV-1 seropositivity or HIV-1 exposure<br>• Severe acute malnutrition<br>• Possible tuberculosis (coughing $\geq$ 14 days)<br>• Anemia with hemoglobin <8·0 g/dL<br>• Severe malaria<br>• Known allergy to penicillin or amoxicillin<br>• Receipt of an antibiotic treatment in the 48 hours prior to the study<br>• Hospitalized within 14 days prior to the study<br>• Living outside the study area<br>• Any medical or psychosocial condition or circumstance that, in the opinion of the investigators, would interfere with the conduct of the study or for which study participation might jeopardize the child's health<br>• Any non-pneumonia acute medical illness which requires antibiotic treatment per local standard of care<br>• Participation in a clinical study of another investigational product within 12 weeks prior to randomization or planning to begin participation during this study<br>• Prior participation in the study during a previous pneumonia diagnosis
**Treatment failure**	
Anytime on or before Day 4	• Severe respiratory distress<br>• Hypoxemia<br>• WHO IMCI danger signs<br>• Missing $\geq$2 study drug doses due to vomiting<br>• Change in antibiotics prescribed by a study clinician<br>• Hospitalization due to pneumonia (if not initially admitted)<br>• Prolonged hospitalization or re-admission due to pneumonia (if initially admitted)<br>• Death
On Day 4 only	• Axillary temperature $\geq$38ºC in the absence of a diagnosed co-infection with fever symptoms (e.g., malaria)
**Relapse**	
Anytime after Day 4	• Recurrence of signs of pneumonia<br>• Signs of severe disease

© 2018 Ginsburg AS et al. *JAMA Pediatrics.*

eAppendix 2. Statistical Analysis Plan

"**Innovative Treatments in Pneumonia (ITIP) 1**:
Double-blind randomized controlled clinical trial of placebo versus 3 days amoxicillin dispersible tablets for fast-breathing childhood pneumonia among children 2-59 months of age presenting to Kamuzu Central Hospital in Lilongwe, Malawi"

Principal Investigator: Amy Ginsburg (Save the Children)
Study Biostatistician: Susanne May (University of Washington)

Statistical Analysis Plan prepared by: Rob Schmicker (University of Washington)
Version 1.0, November 20, 2015
Version 2.0, June 27, 2016
Version 2.1, January 5, 2017

1. INTRODUCTION

Pneumonia is responsible for more than one in five child deaths around the globe. Each year, approximately 1.1 million children die before their fifth birthdays due to pneumonia, more than the number of under-five deaths that result from human immunodeficiency virus (HIV), tuberculosis, and malaria combined. In addition to preventing pneumonia, there is a critical need to provide greater access to appropriate and effective treatment.

Studies in Asia have evaluated the effectiveness of 3 days of oral amoxicillin for the treatment of fast-breathing pneumonia; however, further evidence is needed to evaluate whether any antibiotic treatment is required for the treatment of fast-breathing pneumonia.

The majority of cases diagnosed as fast-breathing pneumonia are not pneumonia at all. There are many possible reasons a child might demonstrate fast-breathing, and not all of them indicate disease. Fast-breathing is neither an appropriately sensitive nor specific sign for pneumonia. Therefore, treatment of fast-breathing with an antibiotic is likely to be unnecessary and inappropriate in the majority of cases, and can lead to the development of antibiotic resistance.

Given the paucity of data from Africa, African-based research is necessary to establish optimal management of childhood pneumonia in the region. With the expressed support of the Malawi Ministry of Health (MOH) and in collaboration with external experts from the University of Washington (UW) Save the Children Federation, Inc. (SC) will work closely with an investigator at the College of Medicine (COM) at the University of Malawi to build evidence regarding whether treatment with amoxicillin dispersible tablets (DT) is necessary for fast-breathing childhood pneumonia in malaria-endemic settings in Africa. An expanded evidence base will contribute to future iterations of integrated community case management guidelines, which in turn will test innovative approaches to childhood pneumonia treatment.

2. ANALYSIS OBJECTIVES

a. Primary Objective
   i. To determine whether treatment with placebo in HIV-negative children 2 to 59 months of age with fast-breathing pneumonia is as effective as 3 days of treatment with oral amoxicillin DT.

b. Secondary Objectives
   i. To determine whether the intervention arm has equivalent rates of treatment relapse as the control arm among those without treatment failure before or on day 4.
   ii. To determine whether the intervention arm has equivalent rates of combined treatment failure and relapse before or on day 14 as the control arm.
   iii. To investigate whether there may be a differential treatment response in children who test positive for malaria at baseline. This information will be useful to plan further childhood pneumonia and malaria integrated interventions in similar settings.
   iv. To determine whether there is a differential treatment response in enrolled children with wheeze during screening (identified prior to any bronchodilator administration).
   v. To determine whether there is a differential treatment response by age.

3. DESIGN

This project involves a double-blinded, randomized, non-inferiority trial in children 2-59 months of age from a malaria-endemic setting in Malawi comparing the effectiveness of placebo to 3-day amoxicillin DT treatment for fast-breathing, community-acquired pneumonia (ITIP1).

We plan to evaluate placebo versus 3 days of oral amoxicillin DT treatment among 2,000 children presenting with fast-breathing pneumonia in a malaria-endemic region of Malawi. Our study will be evaluating twice-daily administration of amoxicillin DT based on age bands (500 mg/day for children 2 months up to 12 months, 1000 mg/day for children 12 months up to 3 years, and 1,500 mg/day for children 3 years up to 5 years of age), the current WHO-recommended therapy.

4. DATA SOURCE

All study data will be collected by the clinical study staff using designated source documents or paper-based case report forms (CRFs). Study data will be entered directly into the CRFs during a study visit. Data from the paper-based CRFs will be entered after the fact into the electronic database as promptly as is feasible.

Clinical research data will be maintained through a combination of secure electronic data management system and physical files with restricted access. Triclinium Clinical Trial Project Management (Pty) Ltd. will serve as the contract research organization (CRO) and will be responsible for primary data management activities during the trial. Among other responsibilities, the data management activities include data entry and validation, data coding and cleaning and database quality control.

Data related to study endpoints will be extracted from the electronic databases for statistical analysis. Two distinct study databases will be available to extract for statistical purposes: the primary study database with study visit data and a safety database with SAE assessments. Statisticians will not have access to any database with participating children's personally identifiable information. The two study databases containing study endpoint data will identify children only by study identification numbers. Data will be provided by the CRO to the study statisticians on a weekly basis.

5. ANALYSIS SETS/POPULATIONS/SUBGROUPS

Children age 2-59 months who present to Kamuzu Central Hospital or Bwaila District Hospital in Lilongwe, Malawi with fast-breathing will be screened for participation in the study. Study staff will perform screening procedures to determine eligibility. They will assign a participant identification number and will collect demographic information, medical history and eligibility criteria data. To be eligible for participation in the study, children must meet the following inclusion/exclusion criteria:

5.1 Inclusion Criteria

- Male or female, 2 to 59 months of age.
- History of cough <14 days or difficult breathing with fast-breathing (for children 2 to <12 months of age, ≥50 breaths/minute and for children ≥12 months of age, ≥40 breaths/minute).
- Ability and willingness of child's caregiver to provide informed consent and to be available for follow-up for the planned duration of the study, including accepting a home visit if he/she fails to return to KCH for a scheduled study follow-up visit.

5.2 Exclusion Criteria

- If fast-breathing observed at screening resolves after bronchodilator challenge, among those with wheeze at screening.
- Chest-indrawing.
- Severe respiratory distress (e.g., grunting, nasal flaring, head nodding, or severe chest-indrawing).
- Presence of WHO IMCI danger signs including: lethargy or unconsciousness, convulsions, vomiting everything, or inability to drink or breastfeed.
- Hypoxia (SaO_2 < 90% on room air, as assessed by a Lifebox pulse oximeter).
- Stridor when calm.
- HIV-1 seropositivity or HIV-1 exposure, assessed as follows:
    - An HIV-positive result upon rapid antibody test will exclude any child from this study.

© 2018 Ginsburg AS et al. *JAMA Pediatrics.*

- If a child is less than 12 months or age with a positive rapid test result, the child will be referred to receive additional confirmatory HIV testing (e.g., dried blood spot filter paper test) and follow-up from KCH staff, as per standard of care. Even if the confirmatory HIV testing subsequently shows that child is HIV-negative, he or she will remain excluded from the study.
- If a child is less than 24 months of age and has an HIV-negative result upon rapid antibody test, the child's biological mother's HIV status will need to be assessed. If the mother is HIV-positive, the child will be excluded. If the mother has a documented HIV-negative test result from within the past 6 weeks, the child will be included. If the mother does not have documentation of an HIV-negative test result, she will be tested via rapid antibody testing to determine the child's eligibility for this study.
- If a child is over 24 months of age, an HIV-negative rapid antibody test is required for inclusion in the study.
- Note: If a child has documentation of an HIV-negative test result from within the past 6 weeks, that test result will be used for the child's eligibility assessment according to the algorithm described above.

- Severe acute malnutrition (weight for height/length < -3 SD, mid-upper arm circumference <115 mm, or edema).
- Possible tuberculosis (coughing for more than 14 days).
- Severe anemia, classified by WHO pocketbook guidelines (i.e., severe palmar pallor or hemoglobin <8.0 g/dL).
- Severe malaria, classified by WHO pocketbook guidelines (i.e., positive mRDT with any danger sign, stiff neck, abnormal bleeding, clinical jaundice, or hemoglobinuria).
- Known allergy to penicillin or amoxicillin.
- Receipt of an antibiotic treatment in the 48 hours prior to the study based on caregiver's self-report and/or documentation in child's medical record.
- Hospitalized within 14 days prior to the study.
- Living outside Lilongwe urban area, the study catchment area.
- Any medical or psychosocial condition or circumstance that, in the opinion of the investigators, would interfere with the conduct of the study or for which study participation might jeopardize the child's health.
- Any non-pneumonia acute medical illness which requires antibiotic treatment per local standard of care.
- Participation in a clinical study of another investigational product within 12 weeks prior to randomization or planning to begin participation during this study.
- Prior participation in an Innovative Treatments in Pneumonia study during a previous pneumonia diagnosis.

Once a child is enrolled in the study, the staff will collect further data on baseline characteristics, vital signs, vaccination history and additional socio-economic information. After being discharged from the hospital within 2-8 hours of enrollment, caretakers will bring enrolled children in for follow-up visits on the mornings of days 2, 3, 4 and 14. They will receive calls from the study staff on the evenings of days 1, 2 and 3. Upon follow-up, study staff will collect information on medical history since the previous visit, study product adherence and results from the physical exam.

Statisticians will have access to all appropriate study visit data on both enrolled patients and screened but not enrolled patients.

5.3 Subgroup analysis

Though listed in the protocol as secondary endpoints, we will examine the primary endpoint of treatment failure by day 4 in the following specific subgroups:

1. Proportion of children with treatment failure among those testing positive for malaria by rapid diagnostic testing (mRDT) at baseline or outcome assessment (overall).
2. Proportion of children failing treatment among those with wheeze during screening (identified prior to administration of bronchodilators).
3. Proportion of enrolled children failing treatment among those children 2-11 months, 12-35 months, and 36-59 months of age at baseline.
4. Proportion of enrolled children failing treatment among those with oxygen saturation <93% by pulse oximetry at baseline.

© 2018 Ginsburg AS et al. *JAMA Pediatrics.*

5. Proportion of children failing treatment among those with MUAC-defined moderate malnutrition (11.5-13.5cm).
6. Proportion of children failing treatment among those with very fast-breathing for age (≥70 breaths per minute for 2–11 months, ≥60 breaths per minute for 12–59 months)

The same definition as above will be used for these subgroups analysis. Subgroup classification will be obtained from the screening data.

6. ENDPOINTS AND COVARIATES

6.1 Primary endpoint

The primary endpoint will be the proportion of children failing treatment, defined as the development of any of the following before or on day 4:

- WHO IMCI danger signs
- Oxygen saturation < 90% by pulse oximetry
- Chest-indrawing
- Vomiting within 30 minutes of 2 or more doses of study product
- Change in antibiotics prescribed by a study clinician (e.g., switch to a second-line antibiotic or prescription for onset of a co-infection)
- Hospitalization due to pneumonia (if not initially admitted)
- Prolonged hospitalization or readmission due to pneumonia (if initially admitted)
- Death

At day 4 outcome assessment

- Documented axillary temperature ≥ 38 ºC in the absence of diagnosed co-infection with fever symptoms (e.g., malaria)

Covariates for primary analysis: In addition to the treatment variable, the primary analysis will include (be adjusted for) the following covariates: age (2 months up to 12 months, 12 months up to 3 years, 3 years up to 5 years) and site (Bwaila District Hospital and Kamuzu Central Hospital). Of note, these are the same variable that are used for stratifying randomization. In addition, we will adjust for gender, as gender has been shown to be related to pneumonia mortality[1]

6.2 Secondary endpoints

The trial has the following secondary endpoints

1. Proportion of children with clinical relapse between treatment failure assessment and day 14 follow-up visit among all children without treatment failure before or on day 4.
2. Proportion of children with either treatment failure or clinical relapse before or on day 14 (among all randomized children).

7. STATISTICAL METHODOLOGY

7.1 Design

The study will include 2,000 children aged 2 to 59 months. Children will be randomized to receive oral amoxicillin for 3 days (control) or 3 days of placebo (intervention). Treatment will be block randomized (with concealed block size) to ensure a 1:1 ratio of intervention and control. Randomization will be stratified by age group (2 up to 12 months, 12 months up to 3 years, 3 years up to 5 years) and site (Bwaila District Hospital and Kamuzu Central Hospital). We will use a 1.5 relative non-inferiority margin. If the failure rate in the 3 day arm is 7%, the non-inferiority margin will be 10.5% (=7%*1.5). With a 5% failure rate the non-inferiority margin will be 7.5% (=5%*1.5).

7.2 Interim analysis

We plan two formal interim analysis after about one-third and two third of the children have been enrolled. In addition, we will ask the Data Safety Monitoring Board (DSMB) to review safety data after 100 participants have been enrolled and have completed the treatment regimen at the request of the clinical trial review committee (CTRC) of the Pharmacy, Medicines and Poisons Board (PMPB). The seqDesign[2] software was used to determine the appropriate non-inferiority and futility stopping boundaries at each analysis. We assumed a one-sided test with an alpha=0.025, sample size = 1900 (assuming 5% loss to follow-up in each of the arms), a Pocock

© 2018 Ginsburg AS et al. *JAMA Pediatrics.*

design for early inferiority and O'Brien-Fleming for early non-inferiority stopping boundaries and a 1.5 relative non-inferiority boundary.

The maximum true failure rates at both the interim analyses and final analysis for the placebo group for which the 95% CI excludes the non-inferiority margin for various 3-day treatment group failure rates are provided in Table 1 below.

Table 1. Maximum true failure rates for the placebo arm beyond which stopping for early non-inferiority will be considered

3-day failure rate	Non-inferiority bound	Max placebo failure rate, 1st interim analysis	Max placebo failure rate, 2nd interim analysis	Max placebo failure rate, final analysis
4.0%	6.0%	1.3%	3.6%	4.4%
5.0%	7.5%	2.1%	4.8%	5.7%
6.0%	9.0%	3.0%	6.0%	7.0%
7.0%	10.5%	4.0%	7.2%	8.3%
8.0%	12.0%	5.0%	8.5%	9.7%
9.0%	13.5%	6.0%	9.8%	11.0%
10.0%	15.0%	7.1%	11.1%	12.4%

The minimum true failure rates for the placebo group for which futility is observed for various 3-day treatment group failure rates are provided below.

Table 2. Minimum true failure rates for the placebo arm where futility will be considered

3-day failure rate	Non-inferiority bound	Min placebo failure rate, 1st interim analysis	Min placebo failure rate, 2nd interim analysis	Min placebo failure rate, final analysis
4.0%	6.0%	5.8%	4.8%	4.4%
5.0%	7.5%	7.3%	6.2%	5.7%
6.0%	9.0%	8.7%	7.5%	7.0%
7.0%	10.5%	10.2%	8.9%	8.3%
8.0%	12.0%	11.7%	10.3%	9.7%
9.0%	13.5%	13.2%	11.7%	11.0%
10.0%	15.0%	14.6%	13.1%	12.4%

In addition to the maximum true failure rates at the final analysis for the placebo group for which the 95% CI excludes the non-inferiority margin for various 3-day treatment group failure rates, the power to detect the alternate of exactly the same failure rates in both arms is shown in Figure 1 below. Of note, the estimated power is adjusted for a 5% loss to follow-up rate in each of the arms. This is considered conservative with respect to the multiple imputation procedure that will be used to account for missing outcome values.

© 2018 Ginsburg AS et al. *JAMA Pediatrics.*

Figure 1: Fast-breathing pneumonia failure rates

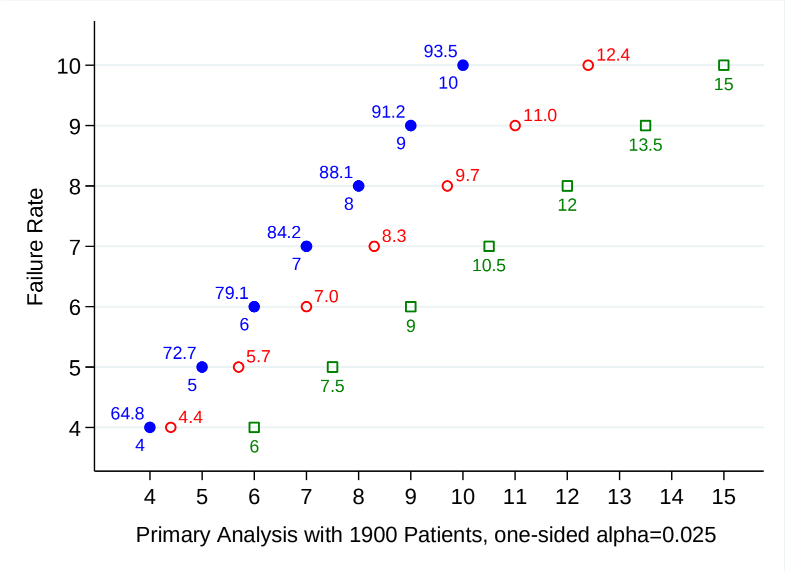

Note:

- Blue, solid circles, blue number below = potential failure rates for the amoxicillin DT treatment group.
- Blue, solid circles, blue number above = Power to detect the alternative of exactly equal failure rates in both treatment groups
- Green, hollow squares = a 1.5 relative non-inferiority margin. For example, for a failure rate of 7%, the non-inferiority margin is 7%*1.5 = 10.5%; for a failure rate of 10%, the non-inferiority margin is 10%*1.5 = 15%.
- Hollow, red circles: maximum true failure rate for the placebo treatment group observed at enrollment of 1,900 children with complete outcome data (950 children in each control and treatment groups) to rule out an increase in failure rate as large as the non-inferiority margin. For example, for a failure rate of 7% in the amoxicillin DT treatment group, the true failure rate in the placebo treatment group can be as large as 8.33% (1.33% above the amoxicillin DT treatment group) and can still rule out a failure rate of 10.5% in the placebo treatment group with a 95% confidence interval at maximum enrollment.

The DSMB will consider recommending to stop the study prior to maximum enrollment if they determine early non-inferiority, early inferiority, or safety concerns.

Early non-inferiority will be considered if the placebo failure rate is lower than the rates provided in Table 1 for a given 3-day treatment failure rate. For instance, if the 3-day treatment rate at the first interim analysis is 5%, then early non-inferiority will be considered at the first interim analysis if the placebo failure rate is less than 2.1%.

Early futility will be considered if the placebo failure rate is higher than the rates provided in Table 2 for a given 3-day treatment failure rate. For instance, if the 3-day treatment rate at the first interim analysis is 6%, then early futility will be considered at the first interim analysis if the placebo failure rate is higher than 8.7%.

Safety concerns that, at a minimum, would necessitate stopping the study prior to maximum enrollment include, but are not limited to: a death in the placebo arm, a significant difference in SAE prevalence in one arm as compared to the other and an overall treatment failure rate in excess of a certain percent of the enrolled population thus far (once at least 100 children have been enrolled). A potentially large treatment failure rate early in the study was a concern of the clinical trial review committee (CTRC) of the Pharmacy, Medicines and Poisons Board (PMPB). In response to their concern the following treatment failure rates will result in stopping of the study after 100 patients have completed their treatment regimen and assessed for treatment failure: if the treatment failure

© 2018 Ginsburg AS et al. *JAMA Pediatrics.*

rate in the placebo arm is 14% or higher and the treatment failure rate in the standard of care (3-day) arm is 7%, (i.e., for doubling of the treatment failure rate). For treatment failure rates in the standard of care arm higher than 7%, a doubling (or more) of treatment failure rate in the placebo arm will lead to stopping the trial. If the treatment failure rate is less than 7%, we will use a slightly revised rule (Figure 2) because for lower treatment failure rates, it becomes more likely to see a doubling in treatment failure rate by chance. For example, the probability is 25% of seeing two or more treatment failures among 50 children (2 failures = 4%) when the true treatment failure rate in that group is actually 2%.

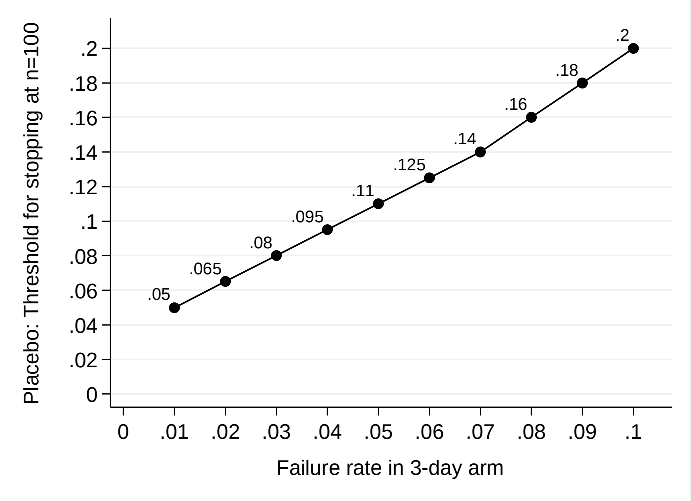

Figure 2: Graphical representation of suggested threshold (solid line) for considering stopping the ITIP1 trial after 100 children (50 in each group) have been enrolled.
The DSMB may require additional, more conservative rules for stopping the study to ensure participant safety.

In addition, the study will be stopped for any death on the placebo arm. Upon notification of a death the ITIP1 study will be halted and, the study pharmacists (local) and statisticians in the United States will then be contacted. The pharmacists and/or statistician will provide the randomization arm for the participant. If the participant is on the placebo arm, the study will be immediately stopped. If the child is found to be in the treatment arm of the study, the death will be treated like all other SAEs.

7.3 Sample Size Increase
There is uncertainty surrounding the true failure rate in the 3-day treatment group. With a fixed sample size of 2000, power = 79% if the 3-day treatment failure rate is 6%. If the actual rate is below this assumption, the power of the study will be below 79% (see Figure above). We will consider the potential to increase the sample size to maintain the pre-specified level of power.

In accordance with FDA guidance on adaptive design in non-inferiority trials[3}, a blinded examination of the overall outcome rate, as opposed to an unblinded examination of the outcome rate in the control group, will not introduce any bias. We will calculate the overall treatment failure rate at time points prior but close to the planned interim analyses to avoid introducing bias.

At the formal interim analysis, we will consider increasing the sample size to maintain at least 80% power (Table 3).

© 2018 Ginsburg AS et al. *JAMA Pediatrics.*

Table 3. Required total sample sizes (mostly rounded to full 100s) for lower failure rates in the amoxicillin DT group to achieve 80%, 85% and 90% power for the alternative of exactly the same failure rates in the two groups (ITIP1)

Overall Failure Rate	80%	85%	90%
4%	3100	3700	4400
4.5%	2800	3200	3900
5%	2500	2900	3500
5.5%	2250	2600	3100
6%	2000	2400	2900
6.5%		2200	2600
7%		2000	2400
7.5%			2300

If the overall treatment failure rate is exceedingly low, increasing the sample size may not be preferred. The minimum threshold for continuing the study with the current study design is still to be determined.

7.4 *Final analysis*
For the primary outcome, we will estimate the difference in failure rate between the placebo and 3-day amoxicillin DT treatment groups after adjustment for age, 2 up to 12 months, 12 months up to 3 years, 3 years up to 5 years, site (Bwaila District Hospital and Kamuzu Central Hospital) and gender (precision variable[1]). Since estimates are expressed in rate differences we will run a linear regression model with treatment arm as predictor (placebo vs. 3-day), failure rate (yes vs. no) as outcome and age, site and gender as covariates using robust standard errors and calculate a 95% CI. The placebo group will be considered non-inferior to the 3-day treatment group if the upper level of the 95% confidence interval excludes a relative 1.5 (times) increase in the failure rate.

To account for anticipated loss to follow-up or study withdrawal we will use multiple imputations for any missing outcome data. We will then perform sensitivity analyses to assess how the results might change with varying imputation assumptions. More information is provided in #8 below.

8. HANDLING OF MISSING DATA AND OTHER DATA CONVENTIONS

We have estimated that loss to follow-up or study withdrawal will be 5%. Children lost to follow-up cannot be classified as improved or treatment failures at the missed visit. We will use multiple imputations for any missing outcome data. The imputations will be performed separately for each treatment group and cohort using multiple (20) hotdeck imputations and adherence information as well as child’s age, gender, and educational status of the caregiver. Multiple imputation estimates will be combined using Rubin’s approach[4].

Specifically, for each episode that is missing outcome data, we will create a subset of episodes that match exactly on treatment arm, and gender while matching on age category, highest educational level achieved by the mother (none, primary, secondary, tertiary, while combining these categories for categories with less than 5% of participants, if needed, unknown education level will be multiply imputed, independent from outcome imputation) and adherence information (no missing doses, missing 1 2, or 3 doses, missing 4, 5 or 6 doses, unknown dosing information will be conservatively imputed as “not taken” for the placebo arm and similar to patients where dosing is available for the 3-day arm). We will then randomly select one episode from the corresponding subset with complete information and use its values to replace the episode with missing outcome data.

We will repeat this process for each episode missing outcome data and calculate failure rates for both treatment arms using the imputed outcome. We will repeat this 20 times and determine the mean failure rates for each treatment arm. The difference between the mean failure rates will be calculated. Noninferiority bounds as listed in 7.2 will be consulted.

© 2018 Ginsburg AS et al. *JAMA Pediatrics.*

9. SENSITIVITY ANALYSIS

We will perform sensitivity analyses to assess how our results might change if the imputation assumptions are changed in a reasonable way, informing the robustness of the primary analysis result. We will perform complete case analysis as one form of sensitivity analysis.

10. PROGRAMMING PLANS

All data cleaning and programming will be done in R[5].

10.1 DSMB Tables

Data tables will be exported from R to Excel in the form of raw data. Raw data files will then be linked to a formatted Excel file and turned into PDF. Draft DSMB Open Session tables will be sent to appropriate clinical and statistical staff prior to the DSMB meeting and will use dummy randomization for the treatment arms. After finalizing content of DSMB tables, real randomization will then be used for the DSMB meeting.

10.2 Manuscript tables

Data tables will be exported from R to Excel in the form of raw data. Raw data files will then be linked to a formatted Excel file. Appendix A displays tables that will be considered for inclusion in the primary manuscript.

© 2018 Ginsburg AS et al. *JAMA Pediatrics.*

SAS APPENDIX A – Tables and Figures

Table 1: Patient and Clinical Characteristics at Enrollment

	Arm A (n=X)	Arm B (n=X)	Overall (n=X)
Age			
Median (IQR)	xx (xx)	xx (xx)	xx (xx)
2-11 months, n (%)	xx (xx%)	xx (xx%)	xx (xx%)
12-35 months, n (%)	xx (xx%)	xx (xx%)	xx (xx%)
36-59 months, n (%)	xx (xx%)	xx (xx%)	xx (xx%)
Sex			
Male, n (%)	xx (xx%)	xx (xx%)	xx (xx%)
Female, n (%)	xx (xx%)	xx (xx%)	xx (xx%)
Weight (kg)			
Median (IQR)	xx (xx)	xx (xx)	xx (xx)
Height/length (cm)			
Median (IQR)	xx (xx)	xx (xx)	xx (xx)
Weight-for-height Z-scores			
Median (IQR)	xx (xx)	xx (xx)	xx (xx)
Mid-upper arm circumference			
Median (IQR)	xx (xx)	xx (xx)	xx (xx)
Vital Signs, median (IQR)			
Axillary Temperature (°C)	xx (xx)	xx (xx)	xx (xx)
Oxygen Saturation (%)	xx (xx)	xx (xx)	xx (xx)
Respiratory Rate (breaths/min)	xx (xx)	xx (xx)	xx (xx)
Pulse Rate (beats/min)	xx (xx)	xx (xx)	xx (xx)
Caregiver Assessment			
Diarrhea	xx (xx%)	xx (xx%)	xx (xx%)
Fever	xx (xx%)	xx (xx%)	xx (xx%)
Cough	xx (xx%)	xx (xx%)	xx (xx%)
Fast/difficult breathing	xx (xx%)	xx (xx%)	xx (xx%)
Chest-indrawing	xx (xx%)	xx (xx%)	xx (xx%)
Nasal blockage	xx (xx%)	xx (xx%)	xx (xx%)
Runny nose	xx (xx%)	xx (xx%)	xx (xx%)
Poor feeding	xx (xx%)	xx (xx%)	xx (xx%)
Feeling cold to touch	xx (xx%)	xx (xx%)	xx (xx%)
Vomiting	xx (xx%)	xx (xx%)	xx (xx%)
Lethargy	xx (xx%)	xx (xx%)	xx (xx%)

© 2018 Ginsburg AS et al. *JAMA Pediatrics.*

Table 2: Treatment Failure Prior to Day 4

	Arm A (n=X)	Arm B (n=X)	Overall (n=X)
Treatment Failure On or Before Day 4	xx (xx%)	xx (xx%)	xx (xx%)
Reason for Treatment Failure			
WHO Danger Signs	xx (xx%)	xx (xx%)	xx (xx%)
Chest-indrawing			
Hypoxia ($SaO_2 < 90\%$)	xx (xx%)	xx (xx%)	xx (xx%)
Axillary Temperature ≥ 38 in absence of diagnosed co-infection w/ fever symptoms	xx (xx%)	xx (xx%)	xx (xx%)
Change in antibiotics	xx (xx%)	xx (xx%)	xx (xx%)
Hospitalization due to pneumonia			
Prolonged hospitalization or re-admission due to pneumonia	xx (xx%)	xx (xx%)	xx (xx%)
Chest-indrawing	xx (xx%)	xx (xx%)	xx (xx%)
Death	xx (xx%)	xx (xx%)	xx (xx%)

Table 3: Treatment Failure Prior to Day 4 for Pre-Specified Subgroups

	Arm A (n=X)	Arm B (n=X)	Overall (n=X)
Malaria Positive Test, n (%)	xx (xx%)	xx (xx%)	xx (xx%)
Malaria Negative Test, n (%)	xx (xx%)	xx (xx%)	xx (xx%)
Wheeze at screening, n (%)	xx (xx%)	xx (xx%)	xx (xx%)
No wheeze at screening, n (%)	xx (xx%)	xx (xx%)	xx (xx%)
Age 2-11 months, n (%)	xx (xx%)	xx (xx%)	xx (xx%)
Age 12-35 months, n (%)	xx (xx%)	xx (xx%)	xx (xx%)
Age 36-59 months, n (%)	xx (xx%)	xx (xx%)	xx (xx%)

© 2018 Ginsburg AS et al. *JAMA Pediatrics.*

References

1. Marzia Lazzerini, Nadine Seward, Norman Lufesi, Rosina Banda, Sophie Sinyeka, Gibson Masache, Bejoy Nambiar, Charles Makwenda, Anthony Costello, Eric D McCollum, Tim Colbourn, 2016, Mortality and its risk factors in Malawian children admitted to hospital with clinical pneumonia, 2001–12: a retrospective observational study, Lancet Global Health, 4, e57-68.
2. Scott Emerson, John Kittelson and Dan Gillen (2014). RCTdesign: Design, monitoring, and analysis of sequential clinical trials.. R package version 1.1.
3. http://www.fda.gov/downloads/Drugs/Guidances/ucm201790.pdf Accessed Nov 13, 2015.
4. Rubin DB. Multiple Imputation for Nonresponse in Surveys. Hoboken, NJ: Wiley Inc; 1987
5. R Core Team (2013). R: A language and environment for statistical computing. R Foundation for Statistical Computing, Vienna, Austria. URL http://www.R-project.org/.

© 2018 Ginsburg AS et al. *JAMA Pediatrics.*

eAppendix 3. Reasons for Study Ineligibility

	n=166[1]
Severe anemia, classified by WHO pocketbook guidelines	62 (37%)
Severe respiratory distress	27 (16%)
History of cough ≥14 days	23 (14%)
HIV seropositivity or exposure	22 (13%)
Chest-indrawing	16 (10%)
Receipt of an antibiotic treatment in the 48 hours prior to the study	12 (7%)
Prior participation in our study during a previous pneumonia diagnosis	11 (7%)
Any medical or psychological condition or circumstance that would interfere with the conduct of the study or for which study participation might jeopardize the child's health	10 (6%)
Any non-pneumonia acute medical illness which requires antibiotic treatment per local standard of care	9 (5%)
Presence of WHO IMCI danger signs	3 (2%)
Severe acute malnutrition	3 (2%)
Hospitalized within 14 days prior to the study	3 (2%)
Living outside Lilongwe urban area	3 (2%)
Age not within two to 59 months	2 (1%)
If fast-breathing observed at screening resolves after bronchodilator challenge	1 (1%)
Stridor when calm	1 (1%)
Possible tuberculosis	1 (1%)
Severe malaria, classified by WHO pocketbook guidelines	1 (1%)

Data are n (%). WHO=World Health Organization. IMCI=Integrated Management of Childhood Illness.
[1]Children may be ineligible for more than one reason.

© 2018 Ginsburg AS et al. *JAMA Pediatrics.*

eAppendix 4. Posteriori Analysis by Treatment Group

	Known treatment failure on or before Day 4		Difference (Placebo - Amoxicillin)
	Amoxicillin (n=552)	Placebo (n=543)	
Age group 2 to 5 months			
RR 50 to 59	0 / 55 (0%)	5 / 56 (9%)	8.9%
RR 50 to 54	0 / 36 (0%)	5 / 37 (14%)	13.5%
RR 55 to 59	0 / 19 (0%)	0 / 19 (0%)	0.0%
RR ≥60	3 / 29 (10%)	1 / 23 (4%)	-6.0%
Age group 6 to 11 months[1]			
RR 50 to 59	5 / 76 (7%)	7 / 84 (8%)	1.8%
RR 50 to 54	2 / 50 (4%)	3 / 57 (5%)	1.3%
RR 55 to 59	3 / 26 (12%)	4 / 27 (15%)	3.3%
RR ≥60	2 / 28 (7%)	2 / 24 (8%)	1.2%
Age group 12 to 23 months			
RR 40 to 49	3 / 78 (4%)	4 / 73 (6%)	1.6%
RR 40 to 44	1 / 58 (2%)	1 / 44 (2%)	0.5%
RR 45 to 49	2 / 20 (10%)	3 / 29 (10%)	0.3%
RR ≥50	4 / 77 (5%)	7 / 57 (12%)	7.1%
Age group 24 to 35 months			
RR 40 to 49	2 / 63 (3%)	4 / 70 (6%)	2.5%
RR 40 to 44	1 / 43 (2%)	3 / 56 (5%)	3.0%
RR 45 to 49	2 / 20 (10%)	3 / 14 (21%)	11.4%
RR ≥50	0 / 34 (0%)	2 / 45 (4%)	4.4%
Age group 36 to 59 months			
RR 40 to 49	2 / 90 (2%)	4 / 85 (5%)	2.5%
RR 40 to 44	2 / 72 (3%)	4 / 67 (6%)	3.2%
RR 45 to 49	0 / 18 (0%)	0 / 18 (0%)	0.0%
RR ≥50	1 / 21 (5%)	2 / 26 (8%)	2.9%
Pneumococcal conjugate vaccine			
Received 3 doses	12 / 291 (4%)	22 / 309 (7%)	3.0%
Received <3 doses or unknown	10 / 261 (4%)	16 / 234 (7%)	3.0%
Pentavalent vaccine			
Received 3 doses	13 / 294 (4%)	22 / 307 (7%)	2.7%
Received <3 doses or unknown	9 / 258 (4%)	16 / 236 (7%)	3.3%

Data are n (%). RR=respiratory rate. [1]One child 6 to 11 months of age had a RR<50 and is not included in table.

© 2018 Ginsburg AS et al. *JAMA Pediatrics.*

eAppendix 5. Treatment Adherence by Age and Treatment Group

Age	**2 to 11 months**	**12 to 35 months**	**36 to 59 months**	**Overall**
**Amoxicillin, n**	196	255	113	564
Doses administered				
1	5 (3%)	6 (2%)	2 (2%)	13 (2%)
2	4 (2%)	1 (<1%)	0 (0%)	5 (1%)
3	1 (<1%)	3 (1%)	2 (2%)	6 (1%)
4	3 (2%)	1 (<1%)	0 (0%)	4 (1%)
5	9 (5%)	4 (2%)	6 (5%)	19 (3%)
6	174 (89%)	240 (94%)	103 (91%)	517 (92%)
Mean (SD)	5 (1.0)	5.8 (0.9)	5.8 (0.8)	5.8 (0.9)
**Placebo, n**	196	254	112	562
Doses administered				
1	5 (3%)	2 (1%)	0 (0%)	7 (1%)
2	0 (0%)	2 (1%)	0 (0%)	2 (<1%)
3	7 (4%)	8 (3%)	1 (1%)	16 (3%)
4	0 (0%)	2 (1%)	0 (0%)	2 (<1%)
5	11 (6%)	12 (5%)	3 (3%)	26 (5%)
6	173 (88%)	228 (90%)	108 (96%)	509 (91%)
Mean (SD)	5.7 (1.0)	5.8 (0.8)	5.9 (0.3)	5.8 (0.8)

Data are n (%) or mean (SD). SD=standard deviation. Doses are marked as administered if they are confirmed by caregiver report and pill count. Unknown doses do not count as administered.

© 2018 Ginsburg AS et al. *JAMA Pediatrics.*

eAppendix 6. Supplementary Material

In the following, details are provided regarding the statistical analysis plan, multiple imputations, tipping point, and as-treated analyses, and the definition of clinical relapse.

Statistical analysis plan

- *5.3 Subgroup analysis*
  - Subgroup analysis for wheeze: There were only three children with wheeze at enrollment. As a result, no subgroup analysis was performed for wheeze
  - Subgroup analysis for oxygen saturation < 93%: There were no children with oxygen saturation <95% at enrollment in this study.
- *6.1 Primary endpoint*, covariates for primary analysis: Because enrollment initially was done solely at KCH and then shifted to solely at Bwaila, this factor is called phase in the manuscript as it represents two phases of enrollment.
- *7.2 Interim analysis*
  - Pocock design for early inferiority: In the first interim analysis, the boundary for early inferiority was crossed and after reviewing additional data summaries the data safety monitoring board recommended that the study be put on hold and data from all enrolled children be analyzed. After subsequent review of data from all enrolled children, the board recommended to stop the study. Because of this, at the analysis that was performed when additional data was available, the early inferiority boundary was considered non-binding and the primary analysis results are not adjusted for interim monitoring.
  - Futility: See comment above regarding the first formal interim and subsequent analysis.
- 7.4 Final analysis: The primary analysis was conceptualized as and intent-to-treat analysis and an as-treated analysis was considered as a sensitivity analysis, but details of the as-treated analysis were not specified a priori
- 8 Handling of missing data and other data conventions: Multiple imputation models were not adjusted for adherence information, because treatment failure was in almost all cases associated with a change in antibiotics and thus lead to non-adherence to the assigned/randomized treatment arm.

Multiple imputations

There are 12 children in the amoxicillin group and 19 children in the placebo group with missing final outcome data. Multiple hot deck imputations were performed to assess the impact of missing outcome data on the primary analysis. For each child missing the final outcome, a donor pool of children was generated with the same treatment group assignment, age group, gender, and mother's education level. With exact matches on all categorical variables, each child with missing primary outcome data had at least 20 matches (and up to 68; median of 40 matches). For each missing outcome, 20 outcomes were sampled, without replacement, from the outcomes of the children in the donor pool. These imputed outcomes, together with the known data, were used to repeat the regression analysis 20 times. The estimates from the regression model were combined using Rubin's method as described in Andridge and Little, 2010.[1]

Tipping point analysis

A tipping point analysis was performed to investigate whether the observed results and conclusions could change depending on what (theoretically) could have been observed for the 12 children in the amoxicillin group and the 19 children in the placebo group with missing Day 4 data if no primary outcome data were missing. In the most extreme case, if all children missing outcome data in the placebo group were successfully treated, and all of those in the amoxicillin group had treatment failure, the results of the study become inconclusive. That is, in that case, we could no longer conclude that placebo is inferior to amoxicillin, but neither could we conclude non-inferiority (nor that placebo is superior to amoxicillin). In this case, the estimate of the difference in treatment failure rates is 0.009 with 95% CI of -0.019 to 0.037. In this case, the estimated amoxicillin event proportion is 6.0%, and so it would be necessary to rule out a difference of 0·030 to conclude non-inferiority. When less extreme cases are considered, results would become inconclusive if, for example, no treatment failures were observed in the placebo group and either two or three treatment failures were observed in the amoxicillin group. Results would also become inconclusive if one or two treatment failures were observed in the placebo group, and three treatment failures were observed in the amoxicillin group (all among the missing data). Furthermore, if all 260 possible combinations of

© 2018 Ginsburg AS et al. *JAMA Pediatrics.*

proportions are considered for the missing data, the results of the study are inconclusive in 32%. Of note, to observe inconclusive results, observed treatment failure rates among the missing data would need to be in the opposite direction of the treatment failure rates observed among children with non-missing primary outcome data.

As-treated analysis

For the as-treated analysis, children with treatment failure within two hours were excluded (five in the amoxicillin and two in the placebo group). Children were considered 100% adherent if either the caregiver reported that all six amoxicillin doses were administered, or if all doses were administered until treatment failure. For example, if a child had treatment failure on Day 3, they were considered 100% adherent if they received four doses of amoxicillin. A child was considered to be at least 80% adherent if they received five out of six doses of amoxicillin (regardless of which dose was missed). A child was also considered 80% adherent if they received four out of five doses of amoxicillin and had a treatment failure after the fifth dose (regardless of which of the five doses was missed). Children in the placebo group were considered to have received no doses of amoxicillin regardless of the number of doses of placebo they received or missed.

Clinical relapse

For the first six months of the study, clinical relapse information was based on data entries regarding relapse in the clinical database. Subsequently, clinical relapse information was also based on serious adverse event data which were collected in a separate database, and additional child characteristics were obtained regarding clinical relapse. As a result, the rate of clinical relapse is anticipated to be slightly lower within the first six months. Nevertheless, because of randomization, we believe that estimated differences between the groups remain unbiased.

Reference

[1] Andridge RR, Little RJ. A Review of Hot Deck Imputation for Survey Non-response. *Int Stat Rev* 2010; **78**(1): 40-64.

© 2018 Ginsburg AS et al. *JAMA Pediatrics.*
