# Supplementary material for: Placebo vs Amoxicillin for Nonsevere Fast-Breathing Pneumonia in Malawian Children Aged 2 to 59 Months: A Double-blind, Randomized Clinical Noninferiority Trial
Source: JAMA Pediatr. 2018 Nov 12;173(1):21–8. doi: 10.1001/jamapediatrics.2018.3407 (PMC6583426; doi:10.1001/jamapediatrics.2018.3407)
Supplement: Supplement 2. — Trial Protocol [file jamapediatr-173-21-s002.pdf]

Trial protocol

Title: Innovative Treatments in Pneumonia (ITIP) 1

A Study of the Pneumonia Innovations Team

Subtitle: Double-blind randomized controlled clinical trial of amoxicillin DT versus placebo for fast-breathing childhood pneumonia among children 2-59 months of age in Lilongwe, Malawi

Sponsored by:

Save the Children Federation, Inc.

Version 9.0
August 26, 2016

Confidentiality Statement

This document is confidential and is to be distributed for review only to investigators, potential investigators, consultants, study staff, applicable regulatory authorities, and applicable independent ethics committees or institutional review boards. The contents of this document shall not be disclosed to others without written authorization from Save the Children (or others, as applicable), unless it is necessary to obtain informed consent from potential study participants.

TABLE OF CONTENTS

TABLE OF CONTENTS 2
ABBREVIATIONS AND ACRONYMS 4
INVESTIGATORS 5
Eric McCollum, MD 5
Assistant Professor, Department of Pediatrics 5
Johns Hopkins School of MedicinePARTICIPATING INSTITUTIONS 5
EXECUTIVE SUMMARY 7
1 BACKGROUND AND INTRODUCTION 9
2 RATIONALE 11
3 STUDY HYPOTHESIS, OBJECTIVES AND ENDPOINTS 11
□ Study Hypothesis 11
□ Study Objectives 11
□ Study Endpoints 12
4 METHODOLOGY 12
4.1 STUDY DESIGN 12
4.2 STUDY SITE 13
4.3 STUDY POPULATION 13
□ Study Population Overview 13
□ Participant Eligibility 13
□ Inclusion Criteria 14
□ Exclusion Criteria 14
4.4 STUDY PERIOD 15
4.5 SAMPLE SIZE 15
4.6 STUDY PROCEDURES 15
□ Recruitment 16
□ Screening 16
□ Informed Consent 17
□ Enrollment Visit 17
□ Randomization 18
□ Management of Study Participants During Hospitalization 18
□ Follow-Up Visits 18
□ Missed Visits 20
□ Interim Contacts and Visits 20
□ Therapy for Treatment Failure and Clinical Relapse 20
□ Withdrawal and Early Termination 20
□ Study Termination Visit 21
□ Biohazard Containment 21
4.7 STUDY PRODUCTS 21
□ Presentation and Formulation 21
□ Preparation and Administration 22
□ Stability and Storage 22
□ Accountability and Disposal 22
4.8 DATA COLLECTION 23
□ Case Report Forms 23
□ Source Documents 23
4.9 DATA MANAGEMENT 23
□ Data Access 23
□ Data Storage 24
□ External Study Monitoring 24

4.10 SAFETY ASSESSMENTS AND REPORTING 24
□ Safety Monitoring 24
□ Data Safety and Monitoring Board (DSMB) 25
□ Adverse Events 25
□ Serious Adverse Event 25
□ Adverse Event Relationship to Study Product 26
□ Grading Severity of Events 26
□ Safety Reporting 26
□ Study Discontinuation 27
5 STATISTICAL DESIGN AND ANALYSIS 27
5.1 DATA ANALYSIS 27
□ Overview and General Design 27
□ Randomization and Blinding Procedures 27
□ Objectives and Endpoints 27
□ Analytical Methodology for Interim Analyses 30
□ Analytical Methodology for Final Analyses 30
5.2 RESULT PRESENTATION 30
5.3 DISSEMINATION OF RESULTS 31
6 ETHICAL CONSIDERATIONS AND CONSENT 31
□ Principles for Clinical Research 31
□ Institutional Review Boards (IRBs) and Independent Ethics Committees (IECs) 31
□ Informed Consent 31
□ Risks to Participants 32
□ Protection against Risks 32
□ Benefits to Participants 33
□ Participant Confidentiality 33
□ Participant Reimbursement 33
□ Storage of Specimens 34
7 POSSIBLE CONSTRAINTS 34
8 REQUIREMENTS AND TRAINING 34
9 REFERENCES 34
APPENDICES 37
APPENDIX I: SCHEDULE OF STUDY VISITS AND EVALUATIONS 37
APPENDIX II: SAMPLE COLLECTION AND LABORATORY EVALUATIONS 37
APPENDIX III: SAMPLE SIZE CALCULATIONS 38
APPENDIX IV: STUDY REQUIREMENTS AND TRAINING 38
APPENDIX V: STUDY SENSITIZATION/RECRUITMENT SCRIPT 39
5.1 Inclusion Criteria .... **Error! Bookmark not defined.**
5.2 Exclusion Criteria .... **Error! Bookmark not defined.**

ABBREVIATIONS AND ACRONYMS

AE	adverse event
AIDS	Acquired Immunodeficiency Syndrome
BDH	Bwaila District Hospital
BMGF	Bill & Melinda Gates Foundation
CFR	Code of Federal Regulations
CI	confidence interval
COM	College of Medicine
COMREC	College of Medicine Research and Ethics Committee
CRF	case report form
CRO	contract research organization
DSMB	Data and Safety Monitoring Board
DT	dispersible tablets
GCP	Good Clinical Practices
HIV	Human Immunodeficiency Virus
ICF	informed consent form
ICH	International Conference on Harmonisation of Technical Requirements for Registration of Pharmaceuticals for Human Use
ID	identification
IEC	independent ethics committee
IMCI	Integrated Management of Childhood Illness
IRB	institutional review board
ITIP	Innovative Treatments in Pneumonia
KCH	Kamuzu Central Hospital
kg	kilogram
mg	milligram
LAR	legally authorized representative
MOH	Ministry of Health
mRDT	malaria rapid diagnostic test
OPD	outpatient department
OR	odds ratio
PI	principal investigator
RR	risk ratio
SAE	serious adverse event
SC	Save the Children Federation, Inc.
SAP	Statistical Analysis Plan
SOP	standard operating procedure(s)
U.S. FDA	United States Food and Drug Administration
UNC	University of North Carolina
UW	University of Washington
WHO	World Health Organization

Innovative Treatments in Pneumonia 1

INVESTIGATORS

Principal investigator (PI):
Amy Ginsburg, MD, MPH
Senior Advisor, Child Health
Save the Children Federation, Inc.

PI signature ____________________ 05/24/2015 Date

Local PI:
Ajib Phiri, MD
Senior Academic Staff, Department of Pediatrics and Child Health
Malawi College of Medicine

Tisungane Mvalo, MB BS, FC Paed
Consultant Pediatrician and Clinical Research Investigator
University of North Carolina (UNC) Project, Lilongwe Trust Medical Relief Fund, Malawi

Co-investigators:
Susanne May, PhD
Associate Professor, Department of Biostatistics
University of Washington

Norman Lufesi, CO, M.Phil.
Program Manager, Acute Respiratory Infection & Emergency Triage Assessment and Treatment
Malawi Ministry of Health

Eric McCollum, MD
Assistant Professor, Department of Pediatrics
Johns Hopkins School of Medicine

PARTICIPATING INSTITUTIONS

**Sponsor:**	Save the Children Federation, Inc. (SC)<br>501 Kings Highway East, Suite 400<br>Fairfield, CT 06825	
**Monitor:**	Triclinium Clinical Trial Project Management (Pty) Ltd.<br>135 West Street, Suite 400<br>Sandown 2196<br>South Africa	
**Medical Officers:**	Amy Ginsburg, MD, MPH<br>Save the Children Federation, Inc. (SC)<br>501 Kings Highway East, Suite 400<br>Fairfield, CT 06825<br>+1-206-258-1827	Janetta Strauss, MPharmMed<br>Triclinium<br>135 West Street, Suite 400<br>Sandown 2196 South Africa<br>+27-11-883-0205
**Clinical Trial Sites:**	Kamuzu Central Hospital<br>Box 149<br>Lilongwe 265 (Area 33)<br>Malawi<br>+265-1-75-3555	
**Data Center:**	Triclinium Clinical Trial Project Management (Pty) Ltd.	
**Study Operations:**	University of North Carolina (UNC) Project, Lilongwe Trust Medical Relief Fund	
**Statistical Support:**	University of Washington (UW)	
**Local Collaborators:**	Malawi Ministry of Health (MOH)<br>College of Medicine (COM) at the University of Malawi<br>Kamuzu Central Hospital (KCH)<br>Save the Children International – Malawi Country Office	
**Funding Agency:**	Bill and Melinda Gates Foundation (BMGF)	

EXECUTIVE SUMMARY

Problem to be studied: Pneumonia is responsible for more than one in five child deaths around the globe. Each year, approximately 1.1 million children die before their fifth birthdays due to pneumonia, more than the number of under-five deaths that result from human immunodeficiency virus (HIV), tuberculosis, and malaria combined. In addition to preventing pneumonia, there is a critical need to provide greater access to appropriate and effective treatment. Studies in Asia have evaluated the effectiveness of 3 days of oral amoxicillin for the treatment of fast-breathing pneumonia; however, further evidence is needed to evaluate whether any antibiotic treatment is required for the treatment of fast-breathing pneumonia.

The majority of cases diagnosed as fast-breathing pneumonia are not pneumonia at all. There are many possible reasons a child might demonstrate fast-breathing, and not all of them indicate disease. Fast-breathing is neither an appropriately sensitive nor specific sign for pneumonia. Therefore, treatment of fast-breathing with an antibiotic is likely to be unnecessary and inappropriate in the majority of cases, and can lead to the development of antibiotic resistance.

Given the paucity of data from Africa, African-based research is necessary to establish optimal management of childhood pneumonia in the region. With the expressed support of the Malawi Ministry of Health (MOH) and in collaboration with external experts from the University of Washington (UW), Save the Children Federation, Inc. (SC) will work closely with an investigator at the College of Medicine (COM) at the University of Malawi and the University of North Carolina (UNC) Project, Lilongwe Trust Medical Relief Fund to build evidence regarding whether treatment with amoxicillin dispersible tablets (DT) is necessary for fast-breathing childhood pneumonia in malaria-endemic settings in Africa. An expanded evidence base will contribute to future iterations of integrated community case management guidelines, which in turn will test innovative approaches to childhood pneumonia treatment.

Type of research: The proposed approach involves conducting a double-blinded, randomized, non-inferiority trial with the objective to assess the effectiveness of no antibiotic treatment for fast-breathing childhood pneumonia in a malaria-endemic region of Malawi.

Objectives: The primary objective of this study is to determine whether treatment with placebo in HIV-negative children 2 to 59 months of age with fast-breathing pneumonia is as effective as 3 days of treatment with oral amoxicillin DT.

Methodology: The study will enroll 2,000 children presenting to Kamuzu Central Hospital or Bwaila District Hospital in Lilongwe, Malawi. Each child will be randomized to either 3 days of amoxicillin DT or placebo DT and will be followed for 14 days with regular study visits at days 2, 3, 4 and 14 after enrollment.

Expected findings and their dissemination: We predict that the rates of treatment failure will be similar in both arms and that placebo will be non-inferior to 3 days of amoxicillin DT for fast-breathing pneumonia. Findings from this study will be disseminated through a peer-reviewed journal and shared with the scientific community.

PROTOCOL OUTLINE

Title: **Innovative Treatments in Pneumonia (ITIP) 1**: Double-blind randomized controlled clinical trial of 3 days of amoxicillin DT versus placebo for fast-breathing childhood pneumonia among children 2-59 months of age in Lilongwe, Malawi

Sponsor: Save the Children Federation, Inc. (SC)

Collaborating Organizations:
Save the Children International – Malawi Country Office
University of Washington (UW)
Malawi Ministry of Health (MOH)
College of Medicine (COM) at the University of Malawi
University of North Carolina (UNC) Project, Lilongwe Trust Medical Relief Fund
Kamuzu Central Hospital (KCH)

Funding Source: Bill and Melinda Gates Foundation (BMGF)

Study Products: Placebo DT (intervention) vs 3 days (control) of 250 mg amoxicillin DT in two divided doses based on age bands (500 mg/day for children 2 months up to 12 months, 1000 mg/day for children 12 months up to 3 years, and 1,500 mg/day for children 3 years up to 5 years of age)

Rationale: Build evidence regarding whether treatment with amoxicillin DT is necessary for fast-breathing childhood pneumonia in a malaria-endemic setting in Africa

Population: 2,000 HIV-1 seronegative children ages 2-59 months of age with fast-breathing pneumonia

Schema: Eligible volunteers will be randomized in a double-blinded manner in a 1:1 ratio as follows:

ITIP1: fast-breathing

Study groups	N	Day 1	Day 2	Day 3	Day 4	Day 14
Placebo	1,000	X	X	X	X	X
3 days	1,000	X	X	X	X	X

Objectives:
1. Primary: treatment effectiveness
2. Secondary: clinical relapse, treatment failure or clinical relapse, cofactors of malaria, wheeze, and age

Endpoints:
1. Primary:
   - Proportion of children failing treatment
2. Secondary:
   - Proportion of children with clinical relapse
   - Proportion of children with clinical relapse or treatment failure
   - Proportion of children failing treatment among those testing positive for malaria (overall)
   - Proportion of children failing treatment among those with wheeze
   - Proportion of children failing treatment by age

Timeline: Projected duration of enrollment is about 30 months.
All children will be followed for 14 days after randomization.

Innovative Treatments in Pneumonia 1

BACKGROUND AND INTRODUCTION

In resource-limited settings, the World Health Organization's (WHO) integrated management of childhood illness (IMCI) guidelines diagnose pneumonia by identifying fast-breathing.[1] However, the majority of cases diagnosed as fast-breathing pneumonia are likely not pneumonia at all. There are many possible reasons a child might demonstrate fast-breathing, many of which do not indicate disease.[2][3][4] Existing case-management guidelines maximize sensitivity over specificity, resulting in widespread over-prescription of antibiotics. Given the growing problem of antibiotic resistance worldwide, such guidelines must be revised.[5][6] [7]

The current WHO guidelines released in 2014 recognize two classifications of childhood pneumonia. Children with fast-breathing and/or chest-indrawing are considered to have "pneumonia" with outpatient treatment recommended; children with additional symptoms are classified with "severe pneumonia" and the guidelines specify inpatient treatment.[8] Amoxicillin is recommended as the first-line treatment for both subcategories of the "pneumonia" classification.[9][10] In settings of low HIV prevalence, WHO recommendations are for 3 days of twice daily dosing for the children in the fast-breathing subcategory and for 5 days of twice daily dosing for children in the chest-indrawing subcategory.

The WHO recommendations were developed based on a review of the then-existing evidence, using the GRADE methodology.[11] In 2012, WHO published a GRADE evidence table regarding the need to treat fast-breathing pneumonia (called "non-severe pneumonia" at the time) with antibiotics, listing two relevant studies. The expert panel classified this evidence as "low quality" for the use of antibiotics in children with non-severe pneumonia without wheeze. For future updates to the guidelines, further evidence must be generated using carefully designed, scientifically sound studies repeated in multiple geographic regions.

There are methodological issues with some of the research suggesting antibiotics are necessary for fast-breathing. In 2004, WHO panels reviewed data from the NARIMA study, which found high rates of radiographically-confirmed pneumonia among children with fast-breathing in Durban, South Africa and Ho Chi Minh City, Vietnam.[12][13] This study's results were neither peer-reviewed nor published (Dr. Don Thea, personal communication). The sample size of this observational study was relatively small (under 200 children) and did not exclude children with HIV-infection/exposure or severe acute malnutrition, potentially affecting the higher rates of positive radiographs found at the Durban site in particular. Standardization of the study's radiological review was not described in its unpublished report.[14] Other studies have characterized the limitations of chest radiography in children, finding that generating consensus readings is difficult and inconsistent.[15][16]

More recent evidence suggests that antibiotics may not be needed to treat fast-breathing pneumonia as defined by WHO IMCI criteria. However, due to methodological limitations, many studies to date have not provided definitive answers with which to change international guidelines. At a 2014 expert panel meeting at the WHO, multiple experts in the field explicitly called for more research comparing antibiotics to no antibiotics in the management of WHO-defined fast-breathing pneumonia.[17] Indeed, the movement away from antibiotics for community-acquired pneumonia (CAP) is already underway in high-resource settings. In the United States, clinical practice guidelines established by the Pediatric Infectious Diseases Society and the Infectious Diseases Society of America note that for outpatients, "antimicrobial therapy is not routinely required for preschool-aged children with CAP, because viral pathogens are responsible for the great majority of clinical disease. (strong recommendation; high-quality evidence)."[18]

The WHO IMCI diagnosis criteria for pneumonia are widely acknowledged to have low specificity.[19] This lack of specificity results in misclassification in the majority of cases. Fast-breathing may be caused by conditions other than pneumonia, and affected by other factors, such as fever. In multiple studies, children with fast-breathing and cough were usually found to have only mild upper respiratory tract infections and not clinical pneumonia when assessed by physicians or by examining chest radiographs.[2][3][4] An observational study in Pakistan found that out of 1848 children diagnosed with fast-breathing pneumonia, only 14% had radiographical evidence of pneumonia while the rest had either normal chest radiographs (82%) or evidence of bronchiolitis (4%).[2] That study did not identify the laboratory-based etiology of the radiographically-confirmed cases of pneumonia, but the researchers

noted that there were only 26 cases of lobar consolidation (1.4%), a presentation that has been linked to bacterial pneumonia.[20] The rest of the positive chest radiographs had interstitial findings, which are more likely associated with non-bacterial etiologies. A Tanzanian study found that respiratory rate itself is highly dependent on external factors unrelated to disease. Observing 167 children in an outpatient setting, researchers found that the median respiratory rate dropped significantly after the patient spent 60 minutes in a quiet setting, leading to up to 85% misclassification of fast-breathing pneumonia in enrolled infants.[21]

Existing research suggests that the majority of cases of fast-breathing pneumonia will resolve without the use of antibiotics. In a Tanzanian study evaluating the causes of fever among outpatient children, researchers showed that in the absence of critical illness (and after ruling out malaria), most febrile outpatient children can be effectively treated without antibiotics.[22] Studies in South and Southeast Asia found high rates (62% and 85%) of response to bronchodilators among children diagnosed with both fast-breathing pneumonia and wheeze, with low rates (15% and 4%, respectively) of clinical deterioration on follow-up, despite not receiving antibiotics.[23][24] Wheeze has repeatedly been found to be common in viral infections, particularly in those due to respiratory syncytial virus.[25] A 2011 Cochrane Review analysis found no difference in clinical outcomes in cases of bronchiolitis treated with either antibiotics or placebo.[26]

Testing the hypothesis that antibiotics are not needed to manage fast-breathing pneumonia, a double-blind, randomized, equivalence trial in four tertiary hospitals in Pakistan among 900 children aged 2–59 months with WHO-defined fast-breathing pneumonia compared treatment failure after 3 days of oral amoxicillin (45 milligrams [mg]/kilogram [kg]/day) or placebo.[27] The study found that 31 (7.2%) of the 431 children in the amoxicillin arm and 37 (8.3%) of the 442 in the placebo group had therapy failure. This difference was not statistically significant (odds ratio [OR] 0.85; 95% CI 0.50–1.43; P 5 .60). The multivariate analysis identified history of difficult breathing (OR 2.86; 95% CI 1.29–7.23; P 5 .027) and temperature of 37.5ºC at presentation (OR 1.99; 95% CI 1.37–2.90; P 5 .0001) as risk factors for treatment failure by day 5. This research suggests that treatment of fast-breathing with an antibiotic is likely to be unnecessary and inappropriate in the majority of cases.

The consequences of such inappropriate treatment are profound. It is well-established that the overuse of antibiotics leads to the development of antibiotic resistance.[28] Management of fast-breathing cases without antibiotics would be much simpler to do by community health workers and would avoid issues of emerging antibiotic resistance as well as reduce the rates of adverse events (AEs) associated with unnecessary antibiotics.

Currently pneumococcal resistance to amoxicillin is low in African settings; however, the prevalence of bacterial resistance to other antibiotics used to treat pneumonia is on the rise in the region.[29] Indeed, many common pathogens causing childhood respiratory, diarrheal, and sepsis infections are resistant to virtually all first-generation antibiotics as a result of decades of extensive use.[30] In hospitalized Malawian populations, 89-96% of *Streptococcus pneumoniae*—the primary pathogen responsible for bacterial pneumonia—is now resistant to cotrimoxazole.[31][32] These Malawian surveillance studies have identified multiple other antibiotics with appreciable resistance prevalence. Especially as the use of oral amoxicillin increases through the support of WHO and the UN Commission on Life-Saving Commodities for Women and Children, good antibiotic stewardship is increasingly important for amoxicillin to remain a long-term solution for treating childhood pneumonia.[33][34]

In addition to preventing antibiotic resistance, reducing the unnecessary use of antibiotics will decrease the burden on the health systems in resource-limited settings. Rational use of antibiotics would help drive down the costs associated with treatment of childhood pneumonia and allow resources to be allocated to those most likely to benefit from the use of antibiotics. Even though individual doses of amoxicillin are relatively cheap, the overall cost of antibiotic treatment for all children with WHO-defined pneumonia is substantial. In Pakistan, the average cost to treat a single case of childhood pneumonia in an outpatient setting was estimated to be $13.44 in 2006, representing 82% of the country's annual health expenditure per person at the time.[35] The absolute and proportional costs in Southeast Asia are comparable to those in sub-Saharan Africa.[36]

Nor are the effects of antibiotic overuse limited solely to public health consequences. Inappropriate use of antibiotics has risks at the individual level as well. Amoxicillin is known for being relatively well-tolerated among children, but it still has documented side effects. Common AEs include diarrhea, nausea, rash, and vomiting. The list of infrequent side effects for oral amoxicillin includes more severe conditions such as allergic reactions, while rare side effects can be as serious as abnormal liver function tests, interstitial nephritis, seizures, and Stevens-Johnson

syndrome.[37] Exposing children who would not benefit from antibiotics to such AEs should be avoided, when possible. Additionally, there is evidence that early in life, repeated exposure to antibiotics disturbs the gut microbiota in such a way that growth and nutrition can be impaired.[38] This growing body of literature suggests that over the long-term, antibiotic use can result in altered immune processing that may increase the risk of subsequent infections.[39][40] By prescribing antibiotics to children that do not need them, those children have an unnecessarily increased risk of AEs associated with antibiotics and can have long-term deleterious health effects.

Clinical research has long included trials to test established medical practices against a new practice, frequently resulting in medical reversals when an existing practice is found to be no better than a lesser practice.[41] Indeed, clinical trials in children with acute otitis media (AOM) have compared antibiotics to placebo to determine if the standard practice of prescribing antibiotics is warranted. A 2015 meta-analysis of 13 such RCTs from high-income countries concluded that "most cases of AOM spontaneously remit without complications. The benefits of antibiotics must be weighed against the possible harms: for every 14 children treated with antibiotics one child experienced an adverse event (such as vomiting, diarrhea or rash) that would not have occurred had antibiotics been withheld."[42] Amoxicillin is often prescribed for AOM and was used in many of the RCTs reviewed. The acceptable risk-benefit ratio of prescribing amoxicillin is certain to be different for AOM than for fast-breathing pneumonia. Nonetheless, a review of antibiotics for preventing suppurative complications in undifferentiated acute respiratory infections (ARI) concluded that "the quality of evidence currently available does not provide strong support for antibiotic use as a means of reducing risk of otitis media or pneumonia in children up to five years of age with undifferentiated ARIs."[43]

More research is needed and, in particular, more research and evidence is needed from malaria-endemic settings in Africa. In fact, a 2014 Cochrane Review was conducted specifically to investigate the existing evidence comparing antibiotic to no antibiotic treatment for fast-breathing pneumonia. The study found a lack of research in this area and recommended that "trials should be carried out to assess the differences between treatment with antibiotics and no antibiotics for non-severe pneumonia with wheeze in children."[25] A clinical trial, referred to as RETAPP, is currently underway in Karachi, Pakistan comparing 3 days of amoxicillin to placebo for children with fast-breathing pneumonia. Findings from RETAPP and the proposed ITIP1 trial would provide evidence regarding appropriate treatment for fast-breathing pneumonia from South Asia and sub-Saharan Africa. The results of trials from two high pneumonia prevalence settings would provide evidence for updating WHO IMCI guidelines for management of fast-breathing pneumonia globally.

RATIONALE

There is a critical need for African-specific data, as countries in Africa, including Malawi, endeavor to put into place evidence-based policies and treatment guidelines informed by the local context. Many cases of diagnosed "fast-breathing pneumonia" are not pneumonia at all and may be inappropriately treated with antibiotics, leading to the emergence of antibiotic resistance. This blinded study proposes to compare treatment effectiveness of 3 days of amoxicillin versus 3 days of placebo for fast-breathing childhood pneumonia. **Extreme care will be taken in this study to protect the safety of all participants.** The clinical trial described in this protocol is intend to provide evidence that could improve access to care by improving case management of pneumonia at the household level, which will lead to improved drug adherence and significantly reduce childhood pneumonia deaths.

STUDY HYPOTHESIS, OBJECTIVES AND ENDPOINTS

- **Study Hypothesis**

Placebo is non-inferior in terms of treatment effectiveness compared to 3 days of amoxicillin DT treatment for fast-breathing pneumonia.

- **Study Objectives**

The broad objective of this study is to provide scientific evidence assessing the necessity of treatment with amoxicillin DT for fast-breathing childhood pneumonia in a malaria-endemic setting in Malawi, Africa.

  - Primary Objective

        - To determine whether treatment with placebo in HIV-negative children 2 to 59 months of age with fast-breathing pneumonia is as effective as 3 days of treatment with oral amoxicillin DT.

    - Secondary Objectives
        - To determine whether the intervention arm has equivalent rates of treatment relapse as the control arm among those without treatment failure before or on day 4.
        - To determine whether the intervention arm has equivalent rates of combined treatment failure and relapse before or on day 14 as the control arm.
        - To investigate whether there may be a differential treatment response in children who test positive for malaria at baseline. This information will be useful to plan further childhood pneumonia and malaria integrated interventions in similar settings.
        - To determine whether there is a differential treatment response in enrolled children with wheeze during screening (identified prior to any bronchodilator administration).
        - To determine whether there is a differential treatment response by age.

- **Study Endpoints**

    - Primary Endpoints

      Proportion of children failing treatment, defined as the development of any of the following during the specified time periods:

      Any time before or on day 4:
        - WHO danger signs
        - Oxygen saturation < 90% by pulse oximetry
        - Chest-indrawing
        - Vomiting within 30 minutes of 2 or more scheduled (i.e., not repeat) dose administrations of study product
        - Change in antibiotics prescribed by a study clinician (e.g., switch to a second-line antibiotic or prescription for onset of a co-infection)
        - Hospitalization due to pneumonia (if not initially admitted)
        - Prolonged hospitalization or readmission due to pneumonia (if initially admitted)

      At day 4 outcome assessment:
        - Documented axillary temperature $\geq$ 38ºC in the absence of diagnosed co-infection with fever symptoms (e.g., malaria)

    - Secondary Endpoints
        - Proportion of children with clinical relapse between treatment failure assessment and day 14 follow-up visit among all children without treatment failure before or on day 4.
        - Proportion of children with either treatment failure or clinical relapse before or on day 14 (among all randomized children).
        - Proportion of children with treatment failure among those testing positive for malaria by rapid diagnostic testing (mRDT) at baseline (overall).
        - Proportion of enrolled children failing treatment among those with wheeze during screening (identified prior to administration of bronchodilators).
    - Proportion of children failing treatment by age at baseline.

METHODOLOGY

STUDY DESIGN

This project involves a double-blinded, randomized, non-inferiority trial in children 2-59 months of age from a malaria-endemic setting in Malawi comparing the effectiveness of placebo to 3-day amoxicillin DT treatment for fast-breathing, community-acquired pneumonia (ITIP1). We plan to evaluate placebo versus 3 days of oral amoxicillin DT treatment among 2,000 children presenting with fast-breathing pneumonia in a malaria-endemic region of Malawi. Our study will be evaluating twice-daily administration of amoxicillin DT based on age bands (500 mg/day for children 2 months up to 12 months, 1000 mg/day for children 12 months up to 3 years, and 1,500 mg/day for children 3 years up to 5 years of age), the current WHO-recommended therapy. [13] Enrolled children will

be followed for 14 days with frequent clinical contact in the first four days and then an exit visit on Day 14. All children will be closely monitored for treatment failure signs and symptoms; any child failing treatment (in either arm) will be hospitalized and treated with second line antibiotics.

STUDY SITE

The Save the Children (SC) PI; the local co-PI, from the College of Medicine (COM) at the University of Malawi and the University of North Carolina (UNC) Project, Lilongwe Trust Medical Relief Fund; and the team of co-investigators will conduct the research at Kamuzu Central Hospital (KCH) in Lilongwe. A 750-bed government facility, KCH is the primary referral hospital for the central region of Malawi, serving a population of approximately 5 million. Up to 30 or 40 children 5 years of age or younger with fast-breathing pneumonia are seen each day in the outpatient department (OPD) at KCH during the peak pneumonia season. The KCH pediatric department alone admits around 22,000 children per year.

One of the major medical training institutions in Malawi, KCH has four full-time on-call pediatricians, two part-time pediatricians, three medical officers, three to six medical interns, 12 full-time clinical officers, and 45 nurses on staff. In the OPD, two to three clinicians with the support of two to three health surveillance assistants manage the triage area. In addition to the triage area, there are also emergency/resuscitation, priority, and low-risk areas in the OPD. The hospital has a high-functioning laboratory unit and a radiology unit that is capable of conducting chest radiographs (including a mobile unit, which is housed in the pediatric ward), ultrasounds, and computed tomography scans.

STUDY POPULATION

- **Study Population Overview**

Although KCH draws from a large catchment area in the central region of Malawi, children eligible for this study are to be from the Lilongwe District. Malawi is ranked 174th in the United Nations Development Programme's human development index, and almost 89% of the working population earns less than $2USD a day.[44] Lilongwe includes large peri-urban settlements with crowded living conditions and without adequate sanitation infrastructure. Overall, the nation's adult literacy rate is 61%. Life expectancy at birth is 55.31 years and the under-five mortality rate is 71 deaths per 1000 children.

Malaria is endemic in Malawi with the highest prevalence of malaria parasitaemia in children between 6 and 36 months of age (60.1%).[45] The incidence of malaria in the area is highest during the rainy season, between December and April each year. Malawi's adult HIV prevalence was estimated to be 10.3% in 2013. There were 170,000 estimated HIV-positive children 14 and younger. HIV is more prevalent in urban communities than rural areas.[46]

We expect study participants to be representative of the ethnic demographics in the area. We anticipate enrolling equal numbers of female and male children for a total participant population of 2,000 volunteers for this trial.

- **Participant Eligibility**

Study participants will be HIV-1 seronegative children 2-59 months of age who present to KCH or Bwaila District Hospital (BDH) with fast-breathing. Volunteer families will be recruited and screened, those whose children are determined to be eligible, based on the inclusion/exclusion criteria, will be enrolled in the study and followed for 14 days. Recruitment, screening and enrollment can occur at KCH or BDH. Hospital observation or admission, and follow-up will occur at KCH. Final eligibility determination will depend on the results of the medical history, clinical examination, appropriate understanding of the study and completion of the consent process.

Case definition of fast-breathing pneumonia:

- Cough <14 days or difficulty breathing
- Respiratory rate $\geq$50 breaths/minute (for children 2 to <12 months of age) or $\geq$40 breaths/minute (for children $\geq$12 months of age)

- **Inclusion Criteria**

  - Male or female, 2 to 59 months of age.
  - History of cough <14 days or difficult breathing with fast-breathing (for children 2 to <12 months of age, ≥50 breaths/minute and for children ≥12 months of age, ≥ 40 breaths/minute).
  - Ability and willingness of children's caregiver to provide informed consent and to be available for follow-up for the planned duration of the study, including accepting a home visit if he/she fails to return to KCH with the child for a scheduled study follow-up visit.

- **Exclusion Criteria**

  - If fast-breathing observed at screening resolves after bronchodilator challenge, among those with wheeze at screening.
  - Chest-indrawing.
  - Severe respiratory distress (e.g., grunting, nasal flaring, head nodding, or severe chest-indrawing).
  - Presence of WHO IMCI danger signs including: lethargy or unconsciousness, convulsions, vomiting everything, or inability to drink or breastfeed.
  - Hypoxia (SaO_2 < 90% on room air, as assessed by a pulse oximeter).
  - Stridor when calm.
  - HIV-1 seropositivity or HIV-1 exposure, assessed as follows:
    - An HIV-positive result upon rapid antibody test will exclude any child from this study.
    - If a child is less than 12 months of age with a positive rapid test result, the child will be referred to receive additional confirmatory HIV testing (e.g., dried blood spot filter paper test) and follow-up from KCH staff, as per standard of care. Even if the confirmatory HIV testing subsequently shows that child is HIV-negative, he or she will remain excluded from the study.
    - If a child is less than 24 months of age and has an HIV-negative result upon rapid antibody test, the child's biological mother's HIV status will need to be assessed. If the mother is HIV-positive, the child will be excluded. If the mother has a documented HIV-negative test result from within the past 3 months, the child will be included. If the mother does not have documentation of an HIV-negative test result, she will be tested via rapid antibody testing to determine the child's eligibility for this study.
    - If a child is over 24 months of age, an HIV-negative rapid antibody test is required for inclusion in the study.
    - Note: If a child has documentation of an HIV-negative test result from within the past 6 weeks, that test result will be used for the child's eligibility assessment according to the algorithm described above.
  - Severe acute malnutrition (weight for height/length < -3 SD, mid-upper arm circumference <115 mm, or edema).
  - Possible tuberculosis (coughing for more than 14 days).
  - Severe anemia, classified by WHO pocketbook guidelines (i.e., severe palmar pallor or hemoglobin <8.0 g/dL).
  - Severe malaria, classified by WHO pocketbook guidelines (i.e., positive mRDT with any danger sign, stiff neck, abnormal bleeding, clinical jaundice, or hemoglobinuria).
  - Known allergy to penicillin or amoxicillin.
  - Receipt of an antibiotic treatment in the 48 hours prior to the study based on caregiver's self-report and/or documentation in child's medical record.
  - Hospitalized within 14 days prior to the study.
  - Living outside Lilongwe urban area, the study catchment area.
  - Any medical or psychosocial condition or circumstance that, in the opinion of the investigators, would interfere with the conduct of the study or for which study participation might jeopardize the child's health.
  - Any non-pneumonia acute medical illness which requires antibiotic treatment per local standard of care.
  - Participation in a clinical study of another investigational product within 12 weeks prior to randomization or planning to begin participation during this study.
  - Prior participation in an Innovative Treatments in Pneumonia study during a previous pneumonia diagnosis.

STUDY PERIOD

Each child will be followed for 14 days after enrollment. Projected duration of enrollment is anticipated to be about 18 months. The high volume of children presenting to the OPD each day with pneumonia is expected to exceed the capacity of study staff to adequately assess each child for this study, so a maximum of 15 children will be enrolled each day. To avoid potential selection bias, each day children will be screened for enrollment in a sequential manner, as much as possible. Children that are not assessed for this study will receive the standard of care at KCH or BDH, which includes 5 days of oral amoxicillin, a case-by-case assessment for hospitalization, and treatment of any co-infections.

The funding for this study is for three years. This period includes the time required to prepare the necessary documents for the study, train all study personnel, initiate the study site, conduct the study and all data collection procedures, clean and analyze the data, and prepare the results for publication and presentation.

SAMPLE SIZE

Refer to Section 5, Statistical Design and Analysis, for more details. With a sample size of 2,000 children (1,000 per treatment group) we aim to rule out a relative increase in failure rate of 50%. The power of the study to show non-inferiority of the placebo group at the final analysis when both treatment groups have exactly the same failure rate depends on the failure rate in the standard treatment group. The estimated power is adjusted for a 5% loss to follow-up rate in each of the arms. For example, with 1,900 children (950 per treatment arm) this study has 85% power to show non-inferiority of the placebo group if the failure rate in both groups is 7% and the non-inferiority margin is a 50% relative increase (absolute non-inferiority margin of 3.5%). For this failure rate in the standard treatment group (of 7%), at the final analysis the 95% confidence interval would exclude 10.5%, even if the failure rate is as larger as 8.33% in the placebo treatment group. With 1,900 children the power of this study to show non-inferiority ranges from 66% if the failure rates in both groups is 4% to 94% if the failure rates in both groups is 10% (assuming a relative non-inferiority margin of 50% (or equivalently a factor of 1.5). Prior to the interim analysis we will be considering the potential to increase the sample size of the study based on a blinded examination of the overall failure rate, to ensure maintaining a pre-specified level of power. Details will be provided in the Statistical Analysis Plan (SAP). We chose a relative non-inferiority margin for the fast-breathing study (rather than an absolute failure rate) because there is higher uncertainty regarding the failure rate for fast-breathing pneumonia and because a more conservative approach to the non-inferiority margin for smaller failure rates was assumed to be acceptable to policymakers and stakeholders for a study of pneumonia that includes a placebo treatment group.

STUDY PROCEDURES

Figure 1. Study Flow Diagram

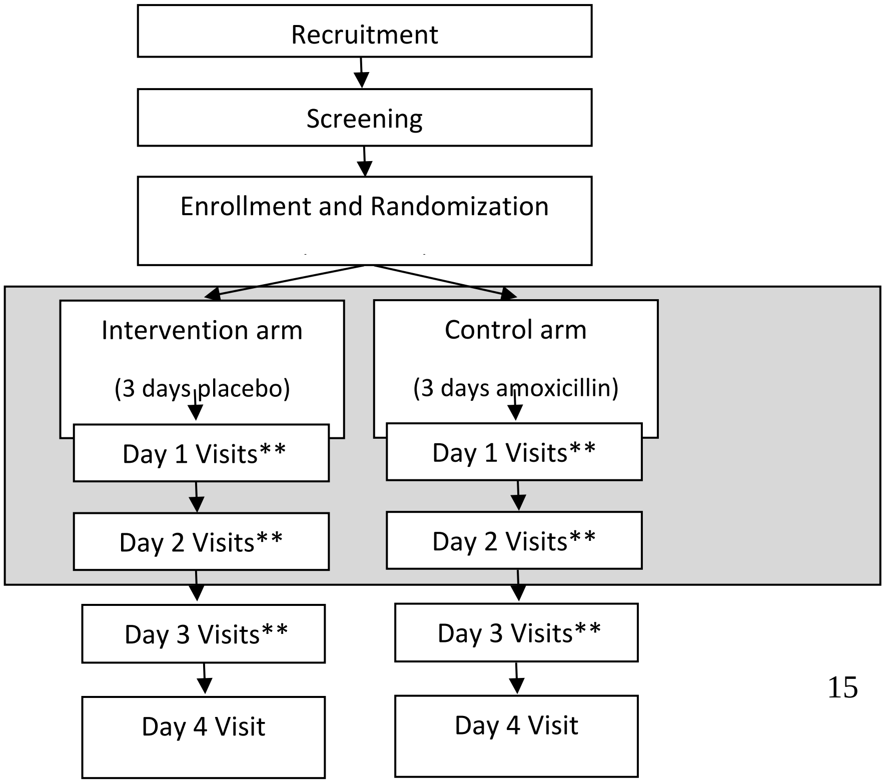

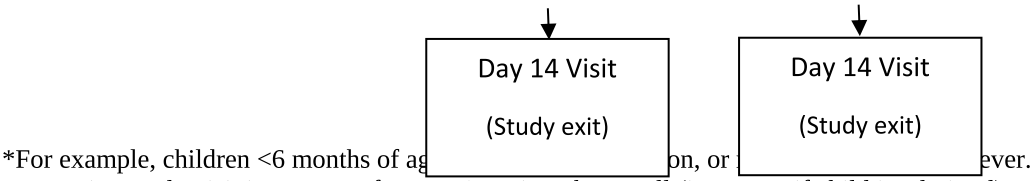

*For example, children <6 months of ag[illegible]on, or [illegible]ever.
**Morning study visit in-person, afternoon/evening phone call (in-person if child is admitted).

Refer to Appendix I for Study Procedures and Visits Table. Refer to Appendix II for Laboratory Specimens Collection, Timing and Distribution Table.

Note that at study initiation, a pilot study will be conducted with up to 100 participants. For these participants, all study procedures as outlined below will be followed EXCEPT for the study product allocation. Pilot participants will undergo mock randomization as all participants in the pilot phase will receive 3 days of active drug. The purpose of this pilot study is to ensure study feasibility, safety and conduct prior to allocating study product based on randomization. Participants in the study pilot will not be informed of the mock randomization process or that they all received active drug. Concealing this will allow study staff to evaluate procedures and participant compliance in a situation that exactly mirrors that of the clinical trial. All data collected on participants enrolled in the pilot study will be entered into a separate database and will not be analyzed with the data from the main trial. The target enrollment of 2,000 participants for the main trial does not include those participants enrolled in the study pilot.

- **Recruitment**

Recruitment for this study will be performed by KCH or BDH staff during routine intake and screening procedures for the OPDs. Children between 2 to 59 months of age presenting to the OPD with cough or difficult breathing will be assessed by hospital staff for potential referral to the study. For any children with a cough fewer than 14 days and fast-breathing, the clinician will read to the caregiver an ITIP recruitment script (refer to Appendix V) with a brief introduction to the study. If the caregiver is interested in learning more about the study and in potentially having the child assessed for eligibility, he/she will be referred to study staff.

All KCH staff involved in recruitment procedures will be trained in relevant study-specific procedures and certified in GCP. Each recruitment and referral interaction will be documented for study records. Due to busy clinic workflow, the study may provide additional staffing assistance in the OPDs, in which case initial recruitment efforts may also be performed by study staff responsible for standard KCH or BDH duties.

- **Screening**

Screening procedures are conducted by study staff to determine eligibility for enrollment in the study. All inclusion/exclusion criteria must be assessed on presentation. The following procedures are performed for screening:

  - Provide information on the study
  - Obtain written informed consent for screening
  - Assign participant identification (ID) number
  - Collect demographic and address information
  - Collect medical history
  - Assess all eligibility criteria, including respiratory rate, chest-indrawing and pulse oximetry assessments (if not already documented in the medical record from that day) as well as a targeted physical examination
  - Perform malaria rapid diagnostic testing (mRDT). Those who are found to have malaria will receive appropriate antimalarial treatment using artemisinin-based combination therapy in addition to the randomly assigned treatment for pneumonia
  - Perform HIV rapid antibody testing if HIV status unknown
  - Perform hemoglobin test (HemoCue®) for anemia

Note that if a child presents with wheezing (audible or auscultatory), study staff will administer a trial of rapid acting inhaled bronchodilator for up to three times, 15-20 minutes apart. Study staff will then assess for fast-breathing and chest-indrawing again to determine the child's eligibility for this study.

All screening procedures will be conducted by study staff, with the possible exception of the HIV rapid antibody test. HIV testing may be performed by either study staff or a team at KCH or BDH specially trained and experienced in pediatric HIV counseling and testing, whichever will reduce wait times for potential study participants and minimize disruption in regular care provision at KCH or BDH. Caregivers will be informed of all screening results during the screening visit, regardless of the eligibility status of their child.

For those children who are not eligible, study staff will inform the caregiver(s) that their child will not be able to participate in the study and will receive standard care at KCH or BDH instead. Children less than 12 months of age or breastfeeding who are excluded based on an HIV-positive rapid antibody test result will be referred for confirmatory testing (e.g., dried blood spot filter paper test).

All screening procedures will be documented in the appropriate study forms, including logs and case report forms. Clinical assessments and findings will also be documented in the child's medical record, as appropriate.

- **Informed Consent**

For the purposes of this protocol, "caregiver" refers to the legally authorized representative (LAR) of the child and informed consent may only be obtained from a child's LAR. Both mother and father are considered LARs for a child, so consent may be obtained from either parent. In the absence of a biological parent, documented proof of legal guardianship would be needed to establish a caregiver's status as a LAR.

This study will have two informed consent forms (ICFs): one for screening procedures and one for enrollment procedures. Informed consent is the process of ensuring that caregivers of children fully understand what will and may happen to their children while participating in a research study. Study staff will administer a comprehension checklist to potential participants' caregivers prior to obtaining written informed consent to ensure that caregivers fully comprehend the nature of the study. The informed consent process continues throughout the study. Key study concepts will be reviewed periodically with the caregivers and the review will be documented. Additionally, if any new information is learned that may affect the caregiver's decision to stay in the trial this information will be shared with the caregivers in writing. All consent materials will be approved by the appropriate Institutional Review Board (IRB) and Independent Ethical Committee (IEC) prior to use.

Refer to detailed description of informed consent procedures and ethical committee approval in Section 6 (Ethical Considerations and Consent).

- **Enrollment Visit**

After screening is complete, study staff will perform the enrollment visit procedures for the trial for only those children who are still eligible. For those children who are eligible, the following procedures are performed for enrollment:

  - Administer comprehension checklist
  - Obtain written informed consent for enrollment
  - Perform a physical exam including vital signs and an assessment of any baseline characteristics not already recorded in the medical record or assessed during screening, including measurement of MUAC
  - Collect vaccination history and additional socio-demographic information
  - Collect locator information to be able to contact caregiver and conduct a home visit, if necessary
  - Follow procedures for randomization assignment
  - Provide the participant caregiver with the appropriate study product kits (described below).
    - A study nurse or clinician will prepare and administer the first drug dose, carefully instructing the caregiver how to administer subsequent doses appropriately. The caregiver will receive an instruction sheet with visual and text descriptions of the timing and dosing necessary to complete the treatment regimen. The pharmacists will be unblinded to the randomization allocation, but both provider and caregiver will be blinded to what the child will receive. The amoxicillin DT and placebo DT will appear and taste the same.
    - Children with fast-breathing will receive amoxicillin DT in two divided doses based on age bands (500 mg/day for children 2 months up to 12 months, 1000 mg/day for children 12

months up to 3 years, and 1,500 mg/day for children 3 years up to 5 years of age) for 3 days (control) or placebo DT in two doses based on age bands for 3 days (intervention).
    - Prescribe concomitant medications, as necessary (e.g., antimalarials if mRDT-positive).

All enrollment procedures will be documented in the appropriate study forms. Clinical assessments and findings will also be documented in the child's medical record, as appropriate.

- **Randomization**

Randomization and enrollment occur at the same study visit, designated Day 1. Randomization is defined as the process of assigning a child to a study arm; assignments are computer-generated by the Protocol Statisticians at UW. The study pharmacists on site will receive the randomization list from the Protocol Statisticians and will be responsible for recording the blinded portion of the randomization code on each study participant's case report forms and blister pack when they are enrolled and randomized. The list that contains a link between the allocation arm and the study participant ID will be maintained by the study pharmacists under lock and key and/or electronic encryption. Other study staff will not have access to the randomization list.

Children will be randomized to placebo (intervention) or 3 days of oral amoxicillin DT (control). Treatments will be allocated in a 1:1 ratio. Study investigators and staff will be blinded to all elements of the randomization allocation for the duration of the study.

- **Management of Study Participants During Hospitalization**

All children enrolled in the fast-breathing study (ITIP1) will be observed in the hospital ward for at least 2-8 hours before being assessed for discharge. Children with no fever and a respiratory rate below the enrolment respiratory rate threshold will be discharged after 2 hours; other children will remain under observation for longer. Children <6 months of age, those with moderate malnutrition (11.5cm-13.5cm MUAC), and those febrile but with a negative mRDT will be initially admitted to the hospital overnight, to be assessed by study staff for discharge on the morning of Day 2.

During discharge assessment, if a child's condition has deteriorated, they will be admitted to the hospital and counted as a treatment failure. Deterioration is defined as: the appearance of any WHO danger sign, oxygen saturation <90%, respiratory rate increased by 10 counts above baseline at enrollment assessment, or appearance of chest-indrawing or other indication of severe pneumonia. During discharge assessment, if a child has an oxygen saturation <93% by pulse oximetry, a respiratory rate above the definition of very fast-breathing, or has developed additional respiratory symptoms, that child will remain in the hospital for continued monitoring. Continued monitoring in hospital will not be treated as prolonged hospitalization unless a child does not meet the criteria for discharge by the morning of Day 3. Once a child does meet the criteria for prolonged hospitalization, that child will be considered to have failed treatment. Note that due to logistical or social factors (e.g., caregiver not present, lack of transport options at that time of day), some children who meet discharge criteria might not leave the hospital right away. When confirmed by a study investigator, such children will not be classified as having prolonged hospitalization and will not qualify as failing treatment, so long as they do not meet any of the other treatment failure criteria.

Children in the study will be primarily managed by study clinicians during any hospitalization, including ward rounds and clinical assessments. Diagnostic tests and medication for intercurrent illnesses will be ordered per ward protocols with results documented in study files. This includes antibiotic treatment regimen changes. To ensure adherence to study protocol, study staff will administer study drugs to participating children during any hospitalization. Study clinicians will be informed by hospital clinicians about the clinical care of children in the study and any clinical decisions made by hospital staff. Study staff will be responsible for orders in the event that study-related laboratory tests and specimen collection are required. The study will be responsible for costs incurred from any laboratory tests performed solely for study purposes. In addition to all study staff, all KCH clinicians and nurses will undergo GCP training.

- **Follow-Up Visits**

Target dates for follow-up visits are calculated from Day 1, the date of randomization. All visits must occur on the calendar day on which they are initially scheduled or within 24 hours afterwards, with the exception for the Day 14 visit, which can occur either 2 days before or after day 14 and still be considered completed within the visit window.

Caregivers will bring their children for follow-up visits: Days 2, 3, 4, and 14. Table 1 highlights the study visits and timing of study product administration.

On Days 1, 2, and 3, children will undergo an in-person visit in the morning and a phone call in the afternoon/evening. The phone call will be conducted by study staff to ascertain the caregiver's assessment of the child's condition and symptoms. Over the phone study staff will also remind caregivers to administer the second dose of study product. If a child is in the hospital during the time period of an afternoon/evening phone visit, the visit will be conducted in-person.

The Day 2 in-person study visit will occur while the child is still hospitalized for those children admitted overnight. Prior to discharge, caregivers will be instructed on how and when to administer the study drug to their child as well as how to contact the study site personnel for concerns that may arise between scheduled visits. Caregivers will receive an instruction sheet with details on the timing and dosing necessary to complete the treatment regimen as well as signs and symptoms that should prompt an immediate call to study staff. A study phone number will be provided to each caregiver and will be answered 24/7.

If a child is not still in the hospital, study staff will attempt to contact the caregiver by phone prior to scheduled study visits to remind them to return to the clinic at the appropriate time.

Table 1. Overview of study follow-up and product administration

	**Day 1**	**Day 2**	**Day 3**	**Day 4**	**Day 14**
**Morning**	• Enrollment visit<br>• Dose 1<br>• Inpatient observation (for all)	• Follow-up visit<br>• Dose 3<br>• Inpatient observation (for select children)	• Follow-up visit<br>• Dose 5	• Follow-up visit	• Follow-up visit and study exit
**Evening**	• Phone call<br>• Dose 2<br>• Inpatient observation (for select children*)	• Phone call<br>• Dose 4	• Phone call<br>• Dose 6		
**All hours**	24-hour hotline number to call				

**Children <6 months of age, with moderate malnutrition (MUAC 11.5cm-13.5cm), or with fever but a negative malaria rapid diagnostic test (mRDT).

Follow-up visit procedures at scheduled in-person visits include the following:

- Review/update locator information
- Review results from prior visits
- Collect medical history since the last study visit
- Perform physical exam including respiratory rate, chest-indrawing and pulse oximetry assessments to assess for treatment failure or clinical relapse
- For all visits EXCEPT Day 14 visit:
  - Collect study product adherence information from caregiver
  - Review drug dosing and administration procedures with caregiver
- At the outcome assessment visit (Day 4):
  - Conduct pill counts of study product and document unused amounts
  - Collect all unused study product from the caregiver

Follow-up visit procedures at scheduled phone call include the following:

- Review/update locator information
- Collect medical history since the last study visit, including adherence information
- Remind caregiver to administer second dose of study product

- Remind caregiver of the next study appointment

For adherence, note that completing 80% of all scheduled dose administrations is considered to meet treatment completion criteria (i.e., 5 out of 6 doses over the 3 days). If doses are missed due to non-adherence, no study action will be taken beyond documenting the missed doses and counseling the caregiver on adherence and study product administration. If a child vomits within 30 minutes of a dose, one repeat dose may be attempted. If a child vomits within 30 minutes after 2 or more scheduled (i.e., not repeat) dose administrations, this will be considered a treatment failure and that child will be referred to care for a work-up of the vomiting cause and will be prescribed a course of second-line antibiotics.

All follow-up visit procedures will be documented in the appropriate study forms. Clinical assessments and findings will also be documented in the child's medical record, as appropriate.

- **Missed Visits**

In case of a no-show at the clinic for a scheduled in-person study visit, study personnel will call the caregiver and visit the child's home either that afternoon or the following day, to conduct the study visit. If study staff is unable to reach a caregiver by phone for a scheduled phone call, at least two repeat attempts will be made in 20 minute intervals. If contact has still not been made with a caregiver, study staff will call the contact(s) that the caregiver listed at enrollment in order to track down the caregiver that same calendar day. Maximum efforts will be made to ensure complete follow-up in the trial. For children who do not complete a scheduled visit within the visit window, that visit will be documented as "missed" but study staff will still attempt to complete the appropriate assessments from that visit, if possible (e.g., Day 4 visit performed and documented on Day 9).

Children who miss a visit, for other than a protocol-mandated reason for discontinuation, are permitted to continue with any subsequent study treatments that can still be scheduled in the time interval specified by the protocol.

Based on our current experience, we expect that fewer than 5% of the children will be lost to follow-up at the time of primary outcome assessment. We think it is unlikely that attrition rates will differ between randomization groups.

- **Interim Contacts and Visits**

Interim contacts and visits (those between regularly scheduled follow up visits) may be performed at caregiver request or as deemed necessary by the site investigators or designee at any time during the study. All interim contacts and visits will be documented in the child's study records and on applicable case report forms (CRF). Interim visits may occur at the study clinic or at the child's home.

Study staff will assess the child for treatment failure or clinical relapse at all interim visits. Study staff will encourage caregivers to call the 24/7 study hotline if they observe any symptoms of concern in their child.

- **Therapy for Treatment Failure and Clinical Relapse**

If a child is determined to have treatment failure on or before Day 4 or clinical relapse between Day 4 and Day 14, he or she will be hospitalized and will receive second-line therapy. At KCH, standard of care for children failing oral amoxicillin is to receive benzyl penicillin and gentamycin. Enrolled children failing treatment or experiencing clinical relapse will receive this regimen as inpatients, regardless of their randomization allocation.

- **Withdrawal and Early Termination**

Children and their caregivers may voluntarily withdraw from the study for any reason at any time. The site investigators may also withdraw children from the study in order to protect their safety if, in the investigators' opinion, continuing participation would jeopardize the child's health. Any participant withdrawal or early termination will be documented in the appropriate study forms.

Any child withdrawn from the study will be referred to care with the recommendation that the child receive a full course of treatment with oral amoxicillin, according to the local standard of care.

- **Study Termination Visit**

The Day 14 visit will serve as the study termination visit for the trial. Procedures for this visit, in addition to the standard follow-up visit procedures described above, include the following:

  - Collect any unused study product from caregiver, if not retrieved at prior study visit
  - Refer child to clinical care, as needed
  - Document contact in child's study records

- **Biohazard Containment**

As exposure to blood-borne pathogens can occur through contact with contaminated needles, blood, and blood products, appropriate blood and secretion precautions will be employed by all personnel in the HIV, anemia, and malaria testing for this study as recommended by the U.S. Centers for Disease Control. Biohazardous waste will be contained according to institutional, transportation/carrier, and all other applicable regulations.

STUDY PRODUCTS

- **Presentation and Formulation**

Prepared study product will be labeled by the manufacturer so as to maintain the blind; both the amoxicillin DT packages and the placebo DT packages will have the same printed information (e.g., "ITIP Study Product - dispersible tablets 250 mg"). This is a double-blinded study in which the study drug assignment will be concealed from the child, caregivers, and study personnel (with the exception of the study pharmacists and the Protocol statisticians). Labels will meet all national and local requirements. The label must also include the product expiry date, batch number, manufacture date.

For this study, amoxicillin DT is supplied as round, orange, uncoated tablets that contain 250 mg of amoxicillin and inactive ingredients. The placebo formulation for this study is a dispersible tablet that appears, tastes, smells, and disperses indistinguishably from the amoxicillin DT, although it is composed of only the inactive ingredients.

Study drug (amoxicillin DT and placebo DT) will be supplied in bulk shipments by a Sponsor-contracted drug manufacturer to the study site. Active drug and placebo pills will be sent in separate shipment packaging to avoid any mis-identification on the part of the study pharmacists, the only un-blinded study staff members in Malawi. The study pharmacists will prepare study product kits in batches based on the stratified randomization list. The amoxicillin DT and placebo DT will be individually labeled by the study pharmacists with each child's participant ID number printed on self-adhesive sticking labels. Different ID sequences and/or label colors will be used for the three age bands to minimize the chances of a prescription error. The link between the participant ID numbers and randomization code will be kept securely by the study pharmacists.

Amoxicillin DT is commercially available in blister packs containing 10 tablets per blister pack. Each child's study product supply will be re-packaged by the study pharmacists in kits based on the child's age band and randomization allocation. Each kit will represent the entire study product supply for one child. A supply of 3 days-worth of study product will be dispensed based on the child's age band (refer to Table 2 below). Each child's kit will consist of either all placebo or all amoxicillin, based on randomization arm.

Table 2. Study Product Kits of Amoxicillin DT or Placebo DT to be taken twice daily

	**No. of tablets provided**
**Age Band**	**Days 1-3**
2 months up to 12 months	6
12 months up to 3 years	12
3 years up to 5 years	18

- **Preparation and Administration**

The amoxicillin DT will be provided in 250 mg doses according to the age bands noted in Table 3 and will be administered orally to the child in divided doses twice daily by dispersing in a small amount of clean water or breast milk.

Table 3. Study Product Administration by Age Band

Age Band	Oral Amoxicillin/Placebo Dispersible Tablets (DT)	
	No. of 250 mg tablets, given two times daily	Total study product administered per day
2 months up to 12 months	1	500 mg
12 months up to 3 years	2	1,000 mg
3 years up to 5 years	3	1,500 mg

Study drug will be maintained and dispensed to the participant caregiver by a study pharmacist. As noted above, the study pharmacists will package tablets received as bulk study drug. Tablets will be used as supplied, meaning that other than packaging and labeling, there is no further study drug preparation required. Study drug is administered orally in either clean water or breast milk, as appropriate. For breast milk administration, the mother will need to express at least 5-10 ml of breast milk into a clean container before dispersing the study product into the liquid. If a mother is unable or unwilling to express milk at the time of study drug administration, clean water can be used instead. For dispersal of study product in water, at least 5-10 ml of bottled, filtered or boiled water can be used in a clean container. Once placed in the liquid, the tablet should be allowed to completely disperse (after at least one minute) before providing the solution to the child to drink. Flavoring agent may be added to the liquid, if desired.

All children, caregivers and research staff will be blinded as to whether the child is in the amoxicillin DT or placebo DT treatment group until the end of the study once the decision to break the study blind is determined by the Sponsor (after completion of study primary manuscript). Codes linking randomization number for each child to actual treatment will be secured in a sealed, opaque envelope and maintained in a locked drawer in the research pharmacy.

Caregivers will be given the emergency contact number for the study personnel during the consenting process in order to report any AEs.

- **Stability and Storage**

All study product not already dispensed to children will be stored in locked cabinets only accessible to the study pharmacists, study clinician, and investigators. The study product does not need to be refrigerated and will be stored in a dry location at ambient room temperatures below 25° C (77° F). Study product will be dispensed to participant caregivers as participant-specific kits by the study pharmacists and documented as such.

- **Accountability and Disposal**

The study pharmacists are required to maintain complete records of all study products received from the Sponsor and/or drug manufacturer and will be responsible for maintaining an accurate record of the randomization codes, inventory, and an accountability record of amoxicillin DT and placebo supplies for this study. The study pharmacists will also be responsible for ensuring the security of these documents, maintaining them under lock and key and/or electronic encryption. Partially used amoxicillin DT and placebo will not be used for human administration.

At the completion of the study, the study pharmacists and site investigators (or designee) will conduct and document a final reconciliation of all study product shipped, received, dispensed, consumed, and remaining. Any discrepancies identified will be investigated, resolved, and documented before any unused study drug is destroyed. After all

accounting and reconciliation procedures are complete and approved by the Sponsor, all unused study product will be destroyed on site and documented in the master study files.

DATA COLLECTION

Clinical research data will be maintained through a combination of secure electronic data management system and physical files with restricted access. Data related to study endpoints will be extracted from the electronic databases for statistical analysis. Three distinct study databases will be created and maintained: the primary study database with study visit data, a safety database with serious adverse event (SAE) assessments, and a database with participating children's personally identifiable information. The first two study databases containing study endpoint data will identify children only by study identification numbers and will not contain identifying information such as name, address, medical record number or personal contact information. In the third database, the study coordinator will maintain a log that will contain the link between personal identifiers and the study participant IDs. The linklog and any other documentation (paper-based or electronic) that has both personal identifiers and the participant ID will have restricted access and will be stored in a secure manner separately from other study data and will be retained for at least five years after the last participating child exits the study.

- **Case Report Forms**

All study data will be collected by the clinical study staff using designated source documents or paper-based case report forms (CRFs). Study data will be entered directly into the CRFs during a study visit. Data from the paper-based CRFs will be entered after the fact into the electronic database as promptly as is feasible. Study staff will maintain source documents for each child at the study site. Source documentation will be available for review to ensure that the collected data are consistent with the CRFs. CRFs and laboratory reports will be reviewed by the site clinical team who are responsible for ensuring that they are accurate and complete. CRFs, source documents and other supporting documents (both electronic and paper-based) will be kept in a secure location and remain separate from participant identification information (name, address, etc.) to ensure confidentiality. Standard Good Clinical Practices (GCP) practices will be followed to ensure accurate, reliable and consistent data collection.

- **Source Documents**

Source documents include but are not limited to:

  - Signed informed consent forms
  - Documentation of the comprehension checklist
  - Visit documentation that includes dates of study visits
  - Receipts for travel reimbursement
  - Reported laboratory results
  - Clinic notes

A copy of all laboratory results will also be included in the child's medical records. Site investigators will maintain, and store in a secure manner, all source documents throughout the study. These documents will be retained for at least five years after the last child exits the study.

DATA MANAGEMENT

Primary data management activities will be undertaken by the designated contract research organization (CRO). The on-site study data manager will oversee data-related procedures at the study site and will be supervised by the CRO data management staff. Data management activities include data entry and validation, data coding and cleaning, database quality control, disaster recovery plans, AE reporting and tracking systems, preparation and submission of safety and compliance reports to the Sponsor, and preparation of final study database. Data management activities will be performed using Clindex® Clinical Trial and Data Management software, developed by Fortress Medical Systems.

- **Data Access**

The participating site will maintain appropriate medical and research records for this trial, in compliance with International Conference on Harmonization Good Clinical Practice E6 (ICH-GCP), regulatory, sponsoring organization and institutional requirements for the protection of confidentiality of children. The site will permit authorized representatives of the Sponsor and regulatory agencies to examine (and when required by applicable law, to copy) clinical records for the purposes of quality assurance reviews, audits and evaluation of the study safety and progress. User-specific usernames and passwords are required to log onto the database. User rights will be provided to study staff, PIs, and co-investigators at the level appropriate for each individual's job description.

- **Data Storage**

The site investigators and designees will maintain, and store securely, complete, accurate and current study records throughout the study. In accordance with regulations, study staff will retain all study records on site for at least five years after study closure. Study records will not be destroyed prior to receiving approval for record destruction from the Sponsor. Applicable records include source documents, site registration documents and reports, informed consent forms, and notations of all contacts with the child.

The Clindex® database is hosted by Fortress Medical Systems through their Software as a Service platform and accessed remotely online. All of the servers that host the Clindex® software and data are housed at ATOMICdata, a Tier 3, SOC 3 Certified Data Center. The primary hosting facility is at the ATOMICdata Minneapolis South facility.

- **External Study Monitoring**

The Study Sponsor and other regulatory authority inspectors or their authorized representatives are responsible for contacting and visiting the study site for the purpose of inspecting the facilities and, upon request, inspecting the various records of the trial. Participant confidentiality will be respected. Site monitoring visits will be conducted to assess compliance with ICH-GCP guidelines. Study monitors will visit the site to:

  - Verify compliance with human subjects and other research regulations and guidelines
  - Assess adherence to the study protocol and study-specific procedures manual
  - Confirm the quality and accuracy of information collected at the study site and entered into the study database
  - Assess the resolution of any past or ongoing issues identified at previous monitoring visits

The site investigators will allow study monitors to inspect study facilities and documentation (e.g., informed consent forms, clinic and laboratory records, other source documents, case report forms), as well as observe the performance of study procedures. Medical records containing identifying information may be made available for review when the study is monitored by the Sponsor or an authorized regulatory agency. Direct access may include examining, analyzing, verifying, and reproducing any records and reports that are important to the evaluation of the study. Site visit logs will be maintained at the study site to document all visits.

SAFETY ASSESSMENTS AND REPORTING

- **Safety Monitoring**

This protocol has extensive safety monitoring in place. The study site investigators will be responsible for close safety monitoring of all children participating in the study, and for alerting the protocol team if unexpected concerns arise. All children will undergo a targeted physical exam at screening and enrollment to ensure that children are medically stable and do not demonstrate any exclusion criteria. Children most at risk for treatment failure will be initially hospitalized overnight to ensure initial continuous and careful monitoring by hospital staff. Each participating child will be evaluated by a study clinician at each in-person study visit. If a child misses an in-person study visit, home visits will be conducted by trained study staff to ensure clinical evaluation. For the first three days of the study, participants will have twice-daily contact with study staff to monitor their health and ensure study product adherence. Every effort will be made to trace all children in the study for the final outcome assessment. An emergency number will be provided to all participants' caregivers so that an on-call clinician can be reached at any time during study participation. As needed, children in the study may be evaluated at interim visits and/or referred for additional care. These "safety net" procedures are intended to identify all instances of potential treatment failure and clinical relapse so that those children failing treatment can be provided appropriate antibiotics and clinical care.

SAEs will also be regularly reviewed by the study's CRO safety monitor and medical expert and compiled into reports for the protocol team. The protocol team may seek independent expert medical opinion as the need arises. In addition, an external group, the Data Safety and Monitoring Board (DSMB) will be closely involved in regular safety monitoring, as described below in more detail.

- **Data Safety and Monitoring Board (DSMB)**

An independent DSMB will be set up to regularly (approximately every 3 months) review cumulative safety and study conduct data. At a minimum, safety data presented to the DSMB will include summaries of data on AEs, SAEs, adherence rates, treatment failure, and clinical relapse. The DSMB will include at least one pediatrician, one pneumonia expert, and one biostatistician. As the interim analyses are available, the DSMB will review interim comparisons of the trial arms after enrollment has begun. The content, format and frequency of safety data reports will be agreed upon by the protocol team and the DSMB in advance of study implementation, to be documented in a DSMB charter. The DSMB may review the unblinded treatment regimens of individuals, if warranted. The DSMB reviews will be summarized with recommendations to the study Sponsor, as to whether or not there are safety concerns and if the study should continue without change, be modified, or terminated.

In the unlikely event that the protocol team has serious safety concerns that lead to a decision to discontinue study product allocation for all children in the study and stop accrual into the study, the protocol team will request an emergency review of the data by the DSMB before recommending that the study be permanently stopped. At the protocol team's request, accrual into the study may be temporarily halted before the DSMB has the opportunity to review the relevant data by treatment arm.

- **Adverse Events**

Per ICH GCP guidelines, an AE is "any untoward medical occurrence in a patient or clinical investigation subject administered a pharmaceutical product and which does not necessarily have a causal relationship with this treatment. An AE can therefore be any unfavorable and unintended sign (including an abnormal laboratory finding), symptom, or disease temporally associated with the use of a medicinal product, whether or not related to the medicinal product." These come to the attention of site clinicians through interim medical histories, physical examinations and laboratory testing. Study participants' caregivers will be instructed to contact the study site staff to report any AEs they may experience. In the case of a life-threatening event, they will be instructed to seek immediate emergency care. Where feasible and medically appropriate, participant caregivers will be encouraged to seek medical care for their children where the study clinician is based, and to request that the clinician be contacted upon their arrival.

All AEs will be managed by the clinical study site team in accordance with good medical practices and the standard clinical practices in place at the hospital. The clinical team will assess and treat or refer the participating child for medical care as appropriate, which may include additional study visits, if necessary. If any acute treatment or medical care is required as a result of harm caused by a study product or study procedure, this care will be provided by the site free of charge. All children in the study with an AE will be followed clinically until the AE resolves (returns to baseline) or stabilizes.

The protocol team anticipates AEs, both severe and non-severe, to occur among enrolled children at a similar rate as untoward medical events occur in comparable pediatric populations outside of a research setting. AEs that the study team expects may occur during the research include, but are not limited to: adverse reactions to amoxicillin (e.g., skin rash), onset of pneumonia-related symptoms (e.g., fever), and onset of other common and uncommon childhood illnesses (e.g., diarrhea, measles). The protocol team expects the vast majority of AEs in this study to be classified as "not related," "probably not related," or "possibly related" to the study product (see section titled "Adverse Event Relationship to Study Product" below).

- **Serious Adverse Event**

SAEs will be defined per US 21 Code of Federal Regulations (CFR) 312.32 guidelines, or the equivalent Malawi regulations, as AEs occurring that:

  - Result in death

- Are life-threatening AEs
- Require inpatient hospitalization or prolongation of existing hospitalization
- Result in persistent or significant disability/incapacity, or
- Are congenital anomalies/birth defects.

Important medical events that may not result in death, be life-threatening, or require hospitalization may be considered serious when, based upon appropriate medical judgment, they may jeopardize the health of the participating child or require medical or surgical intervention to prevent one of the outcomes listed above.

Note that the initial hospitalization for children under 6 months of age, with moderate malnutrition, or with a negative mRDT and fever does not count as an SAE as the condition for which the child was hospitalized occurred prior to administration of the study product, classifying it as a pre-existing condition and not an AE. If that initial hospitalization is prolonged past the expected duration overnight because the child's health has deteriorated, it will be reported as an SAE. Any readmission to the hospital will also be reported as an SAE. Please refer to Section 4.6 on Study Procedures, specifically the portion titled "Management of Study Participants During Hospitalization," for further detail on continued monitoring in hospital and the definition of deterioration.

All treatment failures will be considered SAEs.

- **Adverse Event Relationship to Study Product**

The relationship of all AEs to study product will be assessed as follows:

- Definitely related: AE and administration of study agent are related in time, and a direct association can be demonstrated with the study agent.
- Probably related: AE and administration of study agent are reasonably related in time, and the AE is more likely explained by the study agent than by other causes.
- Possibly related: AE and administration of study agent are reasonably related in time, and the AE can be explained equally well by causes other than the study agent.
- Probably not related: a potential relationship between administration of study agent and AE could exist, but is unlikely, and the AE is most likely explained by causes other than the study agent.
- Not related: the AE is clearly explained by another cause unrelated to administration of the study agent. Reportable events must have documentation to support the determination of "not related."

The assessment for AE relationship to study product must be conducted while the reviewer is blinded to randomization allocation for the child in question.

The initial determination of AE relationship to study product will be made by study staff with as needed consultation with the local PI. An internal medical officer will review determinations of AE relationship and assign the final relationship determination for all Grade 4 and 5 events, including all SAEs. For any death in the study, an independent medical officer will make the final determination of relationship to study product.

- **Grading Severity of Events**

All AEs will be graded by the widely used DAIDS AE Grading Table Version 1.0, December 2004; clarification August 2009. This grading table is now adopted by the U.S. Food and Drug Administration (U.S. FDA) for AE reporting. This table is available at: http://rcc.tech-res.com/Document/safetyandpharmacovigilance/Table_for_Grading_Severity_of_Adult_Pediatric_Adverse_Events.pdf

- **Safety Reporting**

All SAEs must be reported by the site to the medical officers and Sponsor within 24 hours. Attribution with regard to relationship to study product will only be reported for AE grades 2 or above and for all SAEs. Prior to study unblinding, any Grade 4 local or systemic reactogenicity symptom or AE, described by the site staff as possibly, probably, or definitely related to the study product, or any Grade 5 event requires immediate notification by the site to the study coordinator and co-PIs. The co-PIs will convene within 24 hours by teleconference and decide whether

the event necessitates a pause in further enrollment. If the team cannot convene to review the event within 24 hours, the medical officer will make the final decision.

Any death in the study will precipitate immediate action. To be completed within 24 hours, in the following order:

1. Study staff will report the death to the medical officers (internal and independent) and Sponsor.
2. The pharmacists and biostatisticians will be notified of the need for emergency unblinding procedures for that child.
3. If the child is found to be in the placebo arm of the study, enrollment into the study will be automatically stopped.
4. If the child is found to be in the treatment arm of the study, the death will be treated like all other SAEs.

Reporting requirements for the IRB/IEC will be followed as appropriate.

- **Study Discontinuation**

The trial may be discontinued at any time by the protocol team, Sponsor, funding agency, Malawi regulatory authorities, or institutional review board/ethics committee. Please refer to Section 5.1 on Data Analysis, specifically the section on Analytical Methodology for Interim Analyses for more detail on the decision to discontinue the trial for safety.

STATISTICAL DESIGN AND ANALYSIS

DATA ANALYSIS

- **Overview and General Design**

In brief, we plan to conduct a facility-based, double-blinded, individually randomized, non-inferiority trial of placebo (intervention) versus 3 days (control) of oral amoxicillin DT for fast-breathing pneumonia.

The study will include 2,000 HIV-1 seronegative children aged 2 to 59 months presenting with fast-breathing pneumonia at KCH or BDH in Lilongwe, Malawi. The treatment groups will include treatments with placebo two times a day for 3 days versus oral amoxicillin DT in two divided doses for 3 days. Doses will be based on age bands (500 mg/day for children 2 months up to 12 months, 1000 mg/day for children 12 months up to 3 years, and 1,500 mg/day for children 3 years up to 5 years of age). Treatment will be block randomized (with concealed block size) to ensure a 1:1 ratio of intervention and control.

- **Randomization and Blinding Procedures**

Randomization will be performed as 1:1 based on permuted blocks of concealed size within strata defined by age groups (2 up to 12 months, 12 up to 36 months, 36 up to 59 months). Three randomization lists (one for each age group) will be provided by the statisticians to the study pharmacy. The study pharmacists and the Protocol Statisticians will be the only individuals who have access to the treatment assignment for each study patient. Patients, their caregivers and all other study personnel will remain blinded during the course of the study until the primary results manuscript is finalized.

The only type of emergency situation where unblinding a child's randomization allocation would be necessary is in the case of a child death that is "probably not," "possibly," "probably" or "definitely" related to study product. The study pharmacists and biostatisticians will have access to the randomization list to identify a particular child's treatment allocation arm. In this scenario, and any unanticipated need to unblind a child's randomization allocation for reasons of participant safety during the course of the study, the site investigators, Sponsor, and IRB/IEC will be notified and the instance will be documented.

- **Objectives and Endpoints**

The primary null hypothesis will be that the primary outcome of treatment failure at 4 days in those who received placebo is inferior (non-inferior in the alternative hypothesis) to those who received amoxicillin DT when used for 3 days for the treatment of fast-breathing pneumonia in children aged 2–59 months. Eligible children will be randomly assigned to receive placebo twice daily for 3 days in the intervention group and oral amoxicillin DT twice daily for 3 days in the control group. The children will be evaluated on day 4, after 3 days of treatment, to assess treatment failure because by this time point they would have received all their treatment and we would expect them to either be cured or to have failed treatment. They will be evaluated on days 2, 3, and 14 to assess response to treatment. If a child becomes ill again, that child will be encouraged to return between days 4 and 14 to assess for relapse. Children who do not respond to treatment, develop adverse reactions to the study drug, or withdraw from the study will be treated according to Malawian standard guidelines.

The primary outcome will be treatment failure before or on day 4 for intervention and control groups. Secondary outcomes include: (a) clinical relapse between days 4 and 14 if treatment failure was not present before or on day 4, (b) combined rates of clinical relapse and treatment failures before or on day 14, (c) prevalence of malaria among children with cough and/or difficulty in breathing AND fever, (d) differential treatment response among children with wheeze at screening, and (e) differential treatment failure by age. Refer to Section 3 on Hypothesis, Objectives, and Endpoints for full description of all objectives.

- *Treatment failure* will be defined as development of any of the following criteria during the specified time periods:
    - Any time before or on day 4: WHO IMCI danger signs, severe respiratory distress (e.g., grunting, nasal flaring, head nodding, or severe chest-indrawing), oxygen saturation by pulse oximetry < 90%, chest-indrawing, missing 2 or more dose administrations due to vomiting, change in antibiotics prescribed by a study clinician, hospitalization due to pneumonia (if not initially admitted), prolonged hospitalization or re-admission due to pneumonia (if initially admitted), or death.
    - At day 4 outcome assessment: documented axillary temperature $\geq$ 38ºC in the absence of diagnosed co-infection with fever symptoms (e.g., malaria).
    - For the purposes of this protocol, children who do not fail on assessment at day 4 will be considered clinically cured. Loss to follow-up or withdrawal from the study at any time after enrollment and before the day 14 follow-up visit will be considered missing outcome data for that respective time point.
- *Clinical relapse* will be defined as recurrence of signs of pneumonia or severe disease after day 4 among those who did not have treatment failure at or by day 4.

Recruitment and follow-up is expected to continue until the maximum sample size is achieved. We are assuming that a total of 2,000 children (1,000 per treatment group) will be enrolled. We chose a relative non-inferiority margin for the fast-breathing study (rather than an absolute failure rate) because there is higher uncertainty regarding the failure rate for fast-breathing pneumonia and because a more conservative approach to the non-inferiority margin for smaller failure rates was assumed to be acceptable to policymakers and stakeholders for a study of pneumonia that includes a placebo treatment group. Figure 2 shows the maximum true failure rate in the placebo treatment group in reference to various possible failure rates in the amoxicillin DT treatment group and a 1.5 relative non-inferiority margin (50% relative increase in failure rate) that would result in a 95% confidence interval at maximum enrollment which excludes the non-inferiority margin. The figure also indicates the power the study has to show non-inferiority if the failure rates are exactly the same in the two treatment groups for various possible failure rates. Of note, the estimated power is adjusted for a 5% loss to follow-up rate in each of the arms. This is considered conservative with respect to the multiple imputation procedure that will be used to account for missing outcome values.

Figure 2. Fast-breathing pneumonia failure rates

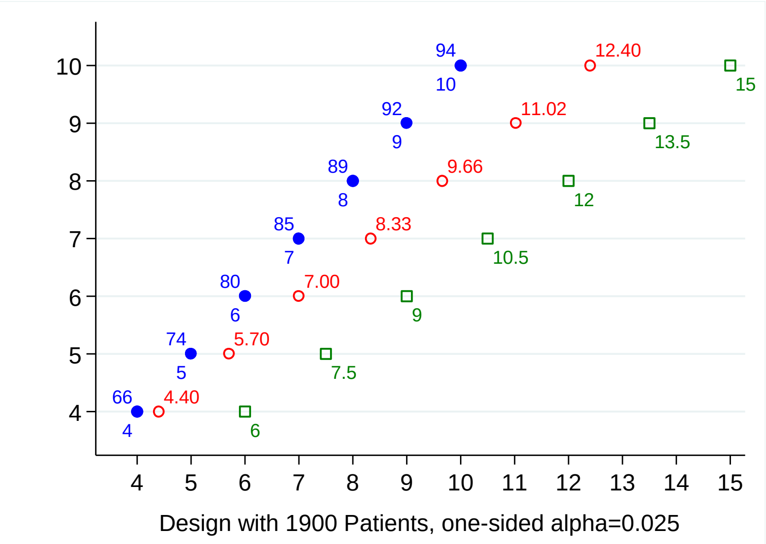

Note:

- Blue, solid circles, blue number below = potential failure rates for the amoxicillin DT treatment group.
- Blue, solid circles, blue number above = Power to detect the alternative of exactly equal failure rates in both treatment groups
- Green, hollow squares = a 1.5 relative non-inferiority margin. For example, for a failure rate of 7%, the non-inferiority margin is 7%*1.5 = 10.5%; for a failure rate of 10%, the non-inferiority margin is 10%*1.5 = 15%.
- Hollow, red circles: maximum true failure rate for the placebo treatment group observed at enrollment of 1,900 children (950 children in each control and treatment groups) to rule out an increase in failure rate as large as the non-inferiority margin. For example, for a failure rate of 7% in the amoxicillin DT treatment group, the true failure rate in the placebo treatment group can be as large as 8.33% (1.33% above the amoxicillin DT treatment group) and can still rule out a failure rate of 10.5% in the placebo treatment group with a 95% confidence interval at maximum enrollment.

Prior to the interim analysis we will be considering the potential to increase the sample size of the study based on a blinded examination of the overall failure rate to ensure maintaining a pre-specified level of power. We will calculate the overall treatment failure rate at a time prior to the planned interim analysis to avoid introducing bias. Table 4 provides potential sample sizes that might be considered in relationship to the failure rate and power of the study. Details of this adaptive design will be provided in the statistical analysis plan (SAP).

Table 4. Required total sample sizes (mostly rounded to full 100s) for lower failure rates in the amoxicillin DT group to achieve 80%, 85% and 90% power for the alternative of exactly the same failure rates in the two groups (ITIP1)

Failure rate in amoxicillin DT group	80%	85%	90%
4%	3100	3700	4400
4.5%	2800	3200	3900
5%	2500	2900	3500

5.5%	2250	2600	3100
6%	2000	2400	2900
6.5%		2200	2600
7%		2000	2400
7.5%			2300

- **Analytical Methodology for Interim Analyses**

We plan one interim analysis after one-half of the children have been enrolled. We assumed a one-sided test with an alpha=0.025, sample size=1900 (assuming 5% loss to follow-up in each of the arms), a Pocock design for early inferiority and O'Brien-Fleming for early non-inferiority stopping boundaries and a 1.5% relative difference failure rate between treatment (placebo) and control (3-day amoxicillin). The DSMB will consider recommending to stop the study prior to maximum enrollment if they determine early non-inferiority, early inferiority or safety concerns.

Safety concerns that, at a minimum, would necessitate stopping the study prior to maximum enrollment include, but are not limited to: a death in the placebo arm with a relationship to study product, overall treatment failure rate in excess of 20% of the enrolled population thus far (once at least 100 children have been enrolled), and a significant difference in SAE prevalence in one arm as compared to the other. The DSMB may require additional, more conservative rules for stopping the study to ensure participant safety.

Details of the sequential monitoring plan will be finalized in collaboration with the DSMB and provided in the Statistical Analysis Plan (SAP).

- **Analytical Methodology for Final Analyses**

We expect the project to produce evidence for fast-breathing pneumonia that supports that placebo treatment is non-inferior to 3 days of amoxicillin DT treatment.

We anticipate that some children may not return for their scheduled follow-up visits if no specific measures are taken to encourage more complete follow-up. In addition to appointment reminders and counseling caregivers on the importance of completing follow-up, study staff will provide incentives and transportation costs to minimize missing outcome data. For those children who do not return for their scheduled follow-up in-person visits, study staff will conduct home visits the next day to assess the outcome. Even with these measures in place, we have estimated the loss to follow-up to be 5% in this study. Children lost to follow-up cannot be classified as improved or treatment failures at the missed visit. We will use multiple imputations for any missing outcome data and perform sensitivity analyses to assess how our results might change if the imputation assumptions are changed in a reasonable way, informing the robustness of the primary analysis result. We will perform complete case analysis as one form of sensitivity analysis. The imputations will be performed separately for each treatment group and cohort using multiple (20) hotdeck imputations and adherence information as well as child's age, gender, literacy status of the caregiver, and number of children in the household.

For the primary outcome, we will estimate the difference in failure rate between the placebo and the 3-day amoxicillin DT treatment groups (after adjustment for age, 2-11 months or 12-59 months) and calculate a 95% CI. The placebo treatment will be considered non-inferior to the amoxicillin DT treatment if the upper level of the 95% confidence interval (CI) excludes a relative increase in failure rate of 50% (factor of 1.5).

To address potential misclassification of eligibility and outcome, we plan to include sub-studies to validate eligibility and outcome in 10% of randomly selected children in the study. For more details on potential misclassification and associated analyses, refer to the SAP.

RESULT PRESENTATION

The results of this research will be primarily presented through at least one published manuscript with detailed description of the background, methods, results, and conclusion. The specific format and details of this manuscript will be in accordance with the requirements of the publishing journal, but is expected to include tables describing the baseline characteristics of study participants and the differences between randomization arms for each study endpoint.

DISSEMINATION OF RESULTS

The results of this study will be published collaboratively by investigators at Save the Children Federation, Save the Children International, University of North Carolina Project, Lilongwe Trust Medical Relief Fund, the University of Washington, the University of Malawi, and the Ministry of Health in peer-reviewed journals. Study findings will be presented to the Malawi MOH Senior Management and hospital staff at the study site. Co-investigators plan on attending at least one international conference to disseminate the findings of the study.

ETHICAL CONSIDERATIONS AND CONSENT

- **Principles for Clinical Research**

This clinical trial will be conducted in compliance with the protocol, International Conference on Harmonization Good Clinical Practice E6 (ICH-GCP), and all applicable regulatory requirements and Institutional Review Boards/Independent Ethics Committee (IRB/IEC) reviews. All study staff will be trained and certified in the protection of human subjects.

Additionally, the protocol team has consulted both the Declaration of Helsinki and the Belmont Report, two cornerstones of ethical principles in human research, while designing this study. The Declaration of Helsinki advises it may be permissible to use less than the standard of care or best proven treatment (e.g., placebo) in certain situations, but that researchers must ensure that patients "will not be subject to additional risks of serious or irreversible harm as a result of not receiving the best proven intervention. Extreme care must be taken to avoid abuse of this option."[47] The Belmont Report emphasizes finding an appropriate balance between the risks and benefits in research.[48] In this study, minimizing the risks to participating children is paramount. Participant safety will be treated as the number one priority through the research, evidenced by a robust safety net for monitoring children in the study, promptly identifying children with treatment failure and getting them the treatment they need to recover; extensive tracing efforts to ensure children do not "slip through the cracks;" and stopping rules that account for a single death in the placebo arm as well as potential safety concerns such as disparate rates of SAEs or higher-than-expected rates of treatment failure.

- **Institutional Review Boards (IRBs) and Independent Ethics Committees (IECs)**

The IRB and IEC of record for this clinical trial are the Western Institutional Review Board (WIRB) and the University of Malawi College of Medicine Research and Ethics Committee (COMREC). A copy of the protocol, proposed informed consent forms, other written participant information, and any proposed advertising material will be submitted to both WIRB and COMREC for written approval. The investigators must submit and, where necessary, obtain approval from the IRB/IEC for all subsequent protocol amendments and changes to the informed consent document. The investigators will notify the IRB/IEC of SAEs according to the IRB/IEC requirements. The Sponsor, CRO, and study operations partner (University of North Carolina Project, Lilongwe Trust Medical Relief Fund) are responsible for assuring that this protocol and the associated informed consent documents and study-related documents are approved by WIRB and COMREC prior to implementation of the protocol. Any amendments to the protocol, informed consents, or other study-related documents must be approved by the IRB/IEC prior to implementation. The study will be conducted in full compliance with the protocol. Any deviations from or violations of the protocol will be documented and submitted to the IRB/IEC by investigators as required. The protocol will not be amended without prior written approval by the PI and Sponsor.

- **Informed Consent**

In obtaining and documenting informed consent, the site investigators and their designees will comply with applicable local and domestic regulatory requirements and will adhere to GCP and to the ethical principles that have their origin in the Declaration of Helsinki. This clinical trial will have an informed consent form (ICF) for screening and an ICF for enrollment developed for local use that are in accordance with all applicable regulations. Both an English and Chichewa version of the ICFs will be reviewed and approved by the IRB/IEC of record before use with participants. The consent forms will include the purpose of the study, the investigational products to be administered, a description of the procedures to be followed and the risks and benefits of participation. The informed consent process will give individuals all of the relevant information they need to decide whether to participate, or to continue participation, in this study. Potential research participants' caregivers will be permitted to ask questions and to exchange information freely with the study team. If the caregiver providing consent is illiterate, an independent witness will be present to verify to the caregiver that all the information read aloud is contained in the ICF. In this instance, both the caregiver and witness will sign the ICF.

Before a child begins participation in the study, it is the site investigators' responsibility to ensure that informed consent is obtained from a LAR after adequate explanation of the aims, methods, and potential risks and benefits of the study. The study staff obtaining consent will also sign and date the ICF. A signed and dated copy of the consent form will be given to the participant's caregiver and this will be documented in the child's record.

- **Risks to Participants**

  - Randomization arms
    This is a randomized trial that is investigating the effectiveness of no treatment with oral amoxicillin DT for pneumonia. It is possible that placebo and amoxicillin are not equivalent in the management of fast-breathing pneumonia and that children receiving placebo could suffer a higher treatment failure rate, with an increased risk of AEs, hospitalization or death. Those children in the amoxicillin arm may have received antibiotics that were unnecessary, exposing them to the potential risks of medication side effects or other microbiota and immune system effects.

  - Coercion
    Caregivers may feel coerced to enroll in the study in order to receive care for their child within a research setting, which may be perceived as of a higher quality than the standard of care.

  - Specimen Collection
    The study involves blood specimen sampling at screening. Phlebotomy can cause pain and bruising at or around the blood draw site.

  - Medical Management
    Participation in the study has the potential to compromise care for hospitalized children, if study procedures are prioritized above urgent clinical care for acute infections.

- **Protection against Risks**

  - Randomization arms
    In order to minimize the risk of AEs, treatment failure, hospitalization, and death, eligibility criteria for this study have been carefully selected and a robust safety monitoring scheme is in place. The children with pneumonia most at risk of treatment failure and/or death will be excluded from this study, including those with WHO IMCI danger signs, severe respiratory distress, HIV infection or exposure, and severe acute malnutrition. Safety monitoring for this study includes frequent clinical examination at study visits for the first four days, outcome assessment and a clinician on call via an emergency hotline for the first fourteen days, treatment and tracking of all AEs and SAEs, and an external DSMB for regular review of cumulative safety and study conduct data. All AEs and SAEs will receive prompt clinical care, as appropriate. Refer to Section 4.10 of this protocol for more detail on the study's Safety Assessments and Reporting procedures.

  - Coercion
    In order to minimize the risk of coercion, study staff will not be recruiting participants directly. Instead, OPD clinicians will inform caregivers about the study and refer only those who are interested. During the

informed consent process, study staff will emphasize that the child will receive medical care whether enrolled in the study or not.

- Specimen Collection
  In order to minimizing the risks associated with phlebotomy, all study staff who will be collecting specimens from children in the study will be trained in the appropriate procedures and supervised accordingly.

- Medical Management
  In order to minimize the possibility that participation in this trial will interfere with the medical management of children with pneumonia at KCH, study staff will have the primary responsibility for the clinical management of hospitalized children. Hospitalized children (e.g., those under 6 months of age during the initial overnight admission) will be treated and managed by study staff in accordance with standard procedures. Study staff will be informed about any decisions regarding treatment failure and changing antibiotic regimens made by KCH staff. The study is prepared to hire additional staff as necessary to avoid overburdening the KCH system. Please refer to Section 4.6 Study Procedures for further description of Management of Study Participants During Hospitalization.

- **Benefits to Participants**

Direct benefits to children in this trial include increased clinical supervision and care during the study period as compared to alternatives not in a study setting. Frequent follow-up visits are not included as standard of care, so participating children will benefit from monitoring for two weeks from the pneumonia episode, including phone calls and home visits for missed follow-up. This level of supervision will make it more likely that a case of treatment failure is identified and managed accordingly as compared to in a non-study setting. Additionally, participants' caregivers will have access to a 24/7 hotline, answered by trained staff, which is not a part of standard of care.

If this trial demonstrates non-inferiority of placebo, the results have great potential to inform and support national and international guidelines for treatment for childhood pneumonia. For example, antibiotics could be prescribed more appropriately, leading to reduced antibiotic resistance and large cost-savings for health systems.

- **Participant Confidentiality**

The site investigators must ensure that the child's confidentiality is maintained. Personal identifiers will not be included in any study reports. All study records will be kept confidential to the extent provided by national and local laws.

All study procedures will be conducted to protect participant privacy and confidentiality to the fullest extent possible. The study site will establish a standard operating procedure (SOP) for confidentiality protection that includes both clinic and home visits and reflects the input of study staff and community representatives to identify potential confidentiality issues and strategies to address them.

- **Participant Reimbursement**

Travel reimbursement will be provided to caregivers to compensate them for the cost of transport for study visits. Reimbursement will be approximately the local currency equivalent of US$5.00 for each scheduled hospital-based study visit, payable at the end of the visit. Reimbursement for interim study visits will be approximately the local currency equivalent of US$2.50. The reimbursement amount may be modified during the course of the study to reflect potential changes in participant costs. The study consent form will list the minimum amount to be paid in the local currency. Participants' caregivers will not receive reimbursement for visits that occur while the child is hospitalized to avoid disruptions in the hospital wards with other non-study patients.

Participants' caregivers will receive a phone card with airtime worth MK 100 on the carrier of their choice (either AirTel or TNM) to cover any phone calls the caregiver may need to make to study staff during the course of the study.

Study participants' caregivers will not be responsible for paying for study-related drugs, tests, or examinations.

- **Storage of Specimens**

Specimens collected during the course of this research will not be stored. Any leftover samples not consumed during study-related diagnostic tests will be destroyed.

POSSIBLE CONSTRAINTS

Anticipated implementation challenges to the successful outcome of the study include:

1. Ensuring quality and consistency of implementation at the trial site. We plan to provide standardized training, supervision, and oversight to ensure quality and harmonized trial procedures. A CRO will be contracted to provide additional oversight and monitoring of the site, as needed.
2. Following up all children. Recognizing that some children may not come back for the follow-up visits, we plan to include and train study staff to locate children who miss their follow-up appointments and conduct these visits in the home. We will also ensure that study staff take the time to educate caregivers on the importance of adhering to the treatment regimen and follow-up.

REQUIREMENTS AND TRAINING

See Appendix VII for a description of study requirements for study activities, including training for study personnel and KCH staff.

REFERENCES

1. World Health Organization. (2014). *Integrated management of childhood illness: chart booklet*. Geneva: World Health Organization.
2. Hazir T, Y. N. (2006). Chest radiography in children aged 2-59 months diagnosed with non-severe pneumonia as defined by World Health Organization: descriptive multi-centre study in Pakistan. *BMJ, 333*(7569), 629.
3. Hazir T, Y. N. (2011). Comparison of oral amoxicillin with placebo for the treatment of World Health Organization-defined nonsevere pneumonia in children aged 2-59 months: a multicentre, double-blind, randomized, placebo-controlled trial in Pakistan. *Clinical Infectious Diseases, 52*(3), 293-300.
4. Mulholland EK, E. S. (1992). Standardized diagnosis of pneumonia in developing countries. *Pediatric Infectious Disease Journal*, *11*, 77-81.
5. World Health Organization. (2013). *Guidelines for the management of common illnesses with limited resources.* Geneva: World Health Organization.
6. English M, Scott JA. What is the future for global case management guidelines for common childhood diseases? *PLoS Medicine.* 2008;5:e241-e241.
7. K, M. (2014). New Diagnostics for Common Childhood Infections. *New England Journal of Medicine, 370*, 875-877.
8. World Health Organization. (2014). *Integrated management of childhood illness: chart booklet*. Geneva: World Health Organization.
9. Grant GB, C. H. (2009). Recommendations for treatment of childhood non-severe pneumonia. *Lancet Infect Dis, 9*, 185-196.
10. Kabra SK, L. R. (2010). Antibiotics for community-acquired pneumonia in children. *Cochrane Database of Systematic Reviews* (3).
11. World Health Organization. (2012). *Recommendations for management of common childhood conditions: evidence for technical update of pocket book recommendations: newborn conditions, dysentery, pneumonia, oxygen use and delivery, common causes of fever, severe acute malnutrition and supportive care.* Geneva: World Health Organization.
12. World Health Organization. (2003 Sep 29-Oct 1). *Consultatitive Meeting to Review Evidence and Research Priorities in the Management of Acute Respiratory Infections (ARI).* Meeting Report, Chapter: "Non-severe

pneumonia: ARI Case Management: Is Antimicrobial Therapy Necessary?." Geneva: World Health Organization.

13. Thea DM, Vy N, Tran AT, Thula S, Qazi S, Fontaine O, and the NARIMA Study Group. A multicentre observational study of clinical outcome following amoxicillin treatment of non-severe acute respiratory tract infection (NARIMA). Global Congress on Lung Health, 32nd World Conference on International Union against Tuberculosis and Lung Disease (IUATLD), Paris, France, 1-4 November 2001. *Int J Tub and Lung Dis* 2001; 5 (Suppl 1):S11.
14. Thea, Donald. A multi-center prospective obseravational study of clinical outcome following amoxicillin treatment of non-severe acute respiratory tract infection. (2001). Unpublished final report.
15. Hopstaken RM, Witbraad T, van Engelshoven JMA, et al. Inter-observer variation in the interpretation of chest radiographs for pneumonia in community acquired lower respiratory tract infections. *Clin Radiol* 2004; **59 :** 743–52.
16. Davies HD, Wang EE, Manson D, et al. Reliability of the chest radiograph in the diagnosis of lower respiratory infections in young children. *Pediatr Infect Dis J* 1996; **15:** 600–04.
17. Sadruddin S. (2014). Save the Children. Toronto. *Personal communication.*
18. Bradley JS, et al. (2011). The management of community-acquired pneumonia in infants and children older than 3 months of age: clinical practice guidelines by the Pediatric Infectious Diseases Society and the Infectious Diseases Society of America. *Clin Infect Dis*, 53(7): e25-76.
19. Rambaud-Althaus, C, et al. (2015). Clinical features for diagnosis of pneumonia in children younger than 5 years: a systematic review and meta-analysis. Lancet Infect Dis, 15: 439-500.
20. Weerakkody Y, Paks M, et al. Lobar pneumonia. *Radiopaedia*. Accessed March 6, 2015. http://radiopaedia.org/articles/lobar-pneumonia
21. Muro F, Mtove G, Mosha N, Wangai H, Harrison N, et al. (2015). Effect of context on respiratory rate measurement in identifying non-severe pneumonia in African children. *Trop Med Int Health*, doi: 10.1111/tmi.12492
22. D'Acremont V, K. M. (2014 Feb 27). Beyond malaria--causes of fever in outpatient Tanzanian children. *New England Journal of Medicine, 370*(9), 809-17.
23. Hazir T, S. Q. (2004). Assessment and management of children aged 1-59 months presenting with wheeze, fast-breathing, and/or lower chest-indrawing; results of a multicentre descriptive study in Pakistan. *Archives of Disease in Childhood, 89*(11), 1049.
24. Lochindarat S, S. Q. (2008). Are we adequately managing children with wheeze using the standard case management guidelines? *Journal of the Medical Association of Thailand, 91*(Suppl 3), 60-8.
25. Lassi ZS, R. K. (2014). Antibiotic therapy versus no antibiotic therapy for children aged two to 59 months with WHO-defined non-severe pneumonia and wheeze. *Cochrane Database of Systematic Reviews,* (5).
26. Spurling GKP, J. D. (2011). Antibiotics for bronchiolitis in children. *Cochrane Database of Systematic Reviews,* (6).
27. Hazir T, N. Y. (2011 Feb 1). Comparison of oral amoxicillin with placebo for the treatment of world health organization-defined nonsevere pneumonia in children aged 2-59 months: a multicenter, double-blind, randomized, placebo-controlled trial in Pakistan. *Clinical Infectious Diseases, 52*(3), 293-300.
28. Low DE, P. M. (2004 Mar). Optimizing antibacterial therapy for community-acquired respiratory tract infectionsin children in an era of bacterial resistance. *Clin Pediatr (Phila), 43*(2), 135-51.
29. Ginsburg AS, L. T. (2013 Dec). Antibiotic non-susceptibility among Streptococcus pneumoniae and Haemophilus influenzae isolates identified in African cohorts: a meta-analysis of three decades of published studies. *Int J Antimicrob Agents, 42*(6), 482-91.
30. Neu HC. (1992). The crisis in antibiotic resistance. *Science, 257*(5073), 1064-1073.
31. Makoka MH, Miller WC, Hoffman IF, et al. (2012). Bacterial infections in Lilongwe, Malawi: aetiology and antibiotic resistance. BMC Infectious Diseases. 12:67.
32. Cornick JE, Harris SR, Perry CM, et al.(2014). Genomic identification of a novel co-trimoxazole resistance genotype and its prevalence amongst Streptococcus pneumonia in Malawi. J Antimicrob Chemother. 69(2):368-74.
33. UN Commission on Life-Saving Commodities for Women and Children. (2012 Sep). *Commissioner's Report.* New York: UN.
34. Rizal A, J. B. (2010). Acute respiratory infection: Boston university's collaborative research work in the last decade. *Indian Pediatrics 47*(1), 19-24.
35. Hussain, H., H. Waters, et al. (2006). "The cost of treatment for child pneumonias and meningitis in the Northern Areas of Pakistan." Int J Health Plann Manage **21**(3): 229-38.

36. Edejer, T. T., M. Aikins, et al. (2005). "Cost effectiveness analysis of strategies for child health in developing countries." BMJ **331**(7526): 1177.
37. Side Effects: List amoxicillin side effects by likelihood and severity. *WebMD*. Accessed March 2, 2015. http://www.webmd.com/drugs/2/drug-1531-3295/amoxicillin-oral/amoxicillin-oral/details/list-sideeffects
38. Peders J, et al. (2006). Factors influencing the composition of the intestinal microbiota in early infancy. *Pediatrics*, 118(2): 511-21.
39. Buffie CG, et al. (2011). Profound alterations of intestinal microbiota following a single dose of clindamycin results in sustained susceptibility to Clostridium difficile-induced colitis. *Infect Immun*, 80(1): 62-73.
40. Bailey MT. (2012). The contributing role of the intestinal microbiota in stressor-induced incrased in susceptibility to enteric infection and systemic immunomodulation. *Horm Behav*, 62(3): 286-94.

\

41. Prasad V, et al. (2013). A decade of reversal: an analysis of 146 contradicted medical practices. *Mayo Clin Proc*, 88(8): 790-798.
42. Venekamp RP, Sanders SL, Glasziou PP, et al. (2015). Antibiotics for acute otitis media in children. Cochrane Database of Systematic Reviews, (6).
43. Alves Galvao MG, et al. (2014). Antibiotics for preventing suppurative complications from undifferentiated acute respiratory infections in children under five years of age. *Cochrane Database of Systematic Reviews* (2).
44. United Nations Development Programme (UNDP). (2014). *Country Profiles, Malawi.* UNDP.
45. F, D. (2009). Epidemiology of Malaria in Malawi. In B. C. Geubbels E, *The Epidemiology of Malawi.* University of Malawi: Division of Community Health, College of Medicine.
46. United Nations AIDS Program (UNAIDS). (2014). *Country Progress Reports, Malawi.* UNAIDS.
47. World Medical Association (WMA). (October 2013). *Declaration of Helsinki: Ethical Principles for Medical Research Involving Human Subjects*. Fortaleza, Brazil: WMA General Assembly.
48. Department of Health, Education, and Welfare. (April 18, 1979). *Ethical Principles and Guidelines for the Protection of Human Subjects of Research*. Washington, DC: The National Commission for the Protection of Human Subjects of Biomedical and Behavioral Research.

PROTOCOL APPENDICES

APPENDIX I: SCHEDULE OF STUDY VISITS AND EVALUATIONS

	Screening	Enrollment (Day 1)	Day 2	Day 3	Day 4	Day 14	Interim visit(s)
Informed Consent	✓	✓					
Comprehension Checklist		✓					
Participant ID	✓	✓	✓	✓	✓	✓	✓
Eligibility Assessment	✓	✓					
Demographics	✓	✓					
Locator Information	✓	✓	✓	✓	✓	✓	✓
Randomization		✓					
Reimbursement		✓	✓	✓	✓	✓	✓
Schedule Next Visit	✓	✓	✓	✓	✓		
Medical History	✓	✓	✓	✓	✓	✓	✓
Targeted Physical Exam	✓	✓	✓	✓	✓	✓	✓

APPENDIX II: SAMPLE COLLECTION AND LABORATORY EVALUATIONS

Specimen for Diagnostic	Screening	Enrollment (Day 1)	Day 2	Day 3	Day 4	Day 14	Laboratory
HIV test	✓						Study site
Anemia test	✓						
Malaria test	✓						

APPENDIX III: SAMPLE SIZE CALCULATIONS

Graphical representation of required total sample sizes (mostly rounded to full 100s) for lower failure rates in the amoxicillin DT group to achieve 80%, 85% and 90% power for the alternative of exactly the same failure rates in the two groups (ITIP1).

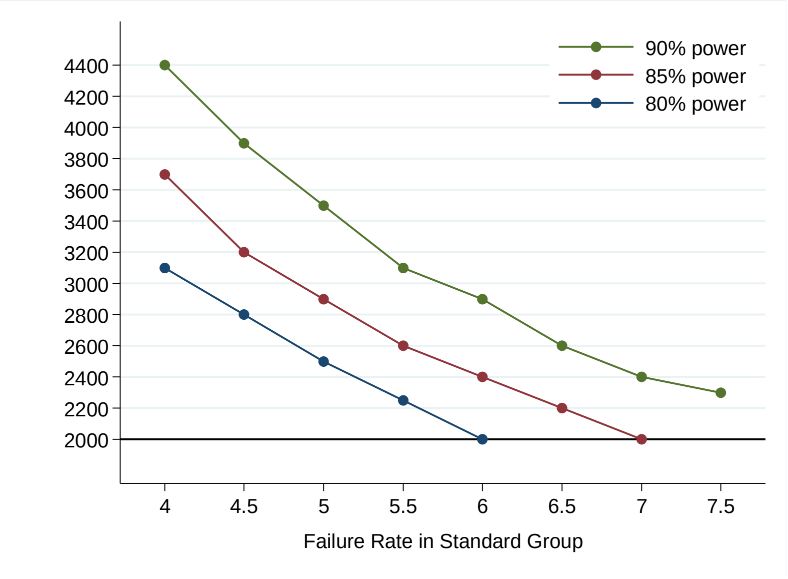

APPENDIX IV: STUDY REQUIREMENTS AND TRAINING

Additional study requirements not already described in the protocol are summarized below.

- **Personnel**

  The study team on-the-ground will consist of full-time employees in the following capacities:
  - Study coordinator: clinician who will oversee daily study operations and monitor the safety of participants
  - Study medical officers/clinical officers: clinicians whose daily work will involve screening potential study participants, enrolling children into the study, performing clinical assessments during observation periods, clinically managing hospitalized study participants, and conducting the safety monitoring and reporting for adverse events in the study patients.
  - Pharmacists: receive, account for, prepare, and distribute study product; train study staff on study product administration procedures
  - Data manager: maintain study databases with quality control and quality assurance procedures; prepare regular reports of ongoing study activities and data
  - Data officers/assistants: scan data collected on paper forms for entry into electronic database(s); maintain copies of study documents as needed, prepare study documents and ensure all in correct format for study participant files, perform quality control checks in the study participants' CRF`s.
  - Study nurses: perform screening, enrollment, follow-up and interim study visit procedures; conduct informed consent process; conduct all home visits and study retention efforts
  - HTC counselors: perform HIV test pre-counseling, testing and post-test counseling per study protocol and Malawi national guidelines.
  - Fieldworkers: perform home follow up visits.

  In addition to the full-time staff, the project will use the expertise of additional Save the Children (SC) personnel to assist with meeting study goals. A portion of SC staff's time will be for operational and grant management services, which will provide a range of support activities to the project such as accounting, human resources management, information technology, administration and audit services.

Government staff at the study site hospital will also be engaged with this study. KCH service providers will be responsible for recruiting of study participants at KCH or BDH and managing participants' care while in the hospital.

- **Training**

All study staff will be trained in the Protection of Human Subjects prior to any interactions with study participants. Additionally, before the study starts, all study staff will attend an extensive 5-day study-specific training to review all study procedures, including the study protocol, SOPs, data collection tools, informed consent process, reporting requirements, and safety monitoring. Refresher trainings on the identification of pneumonia will be scheduled at least once per year and will include updates from the study monitor reports. Trainings will be conducted by a Sponsor representative, representative of the study CRO, or other qualified clinician, as appropriate for the training material.

Government staff at KCH and BDH will be sensitized to this study and will receive at least one day of training on the identification of pneumonia and study-specific procedures and documentation prior to the study start. Refresher trainings will be held periodically, at least once every year.

- **Supplies**

Supplies for this study include the following:

  - Laptops for study staff
  - Printer
  - Photocopier
  - Respiratory rate counters
  - Portable pulse oximeter
  - Scale
  - Height board
  - Malaria RDT kits
  - Office furniture
  - Partitions/privacy screens for the study clinic
  - Communication equipment such as cellphone accessories, airtime, and internet sticks
  - Standard office supplies, including binders, paper, pens

- **Transportation**

The study will obtain multiple motorcycles for use by the study retention team to conduct home visits after a participant misses a scheduled study visit. Study participants will be expected to provide their own transportation to study visits at KCH, but will receive a travel reimbursement.

- **Space**

The study clinic for out-patient screening and enrollment will be located in the Pediatric Department of KCH or BDH. The study clinic for follow-up and interim visits will be located in the Pediatric Department of KCH. The hospital has provided the study with a private room for study visits and other study-related activities. Additional office space for data management and the study coordinator will be provided at a separate location in Lilongwe.

APPENDIX V: STUDY SENSITIZATION/RECRUITMENT SCRIPT

Instructions: This script is to be used by Kamuzu Central Hospital and Bwaila District Hospital staff in the Paediatric Outpatient Department after the initial triage and intake of a presenting child. This content should be presented to caregivers of children who are between 2 and 59 months of age and have cough or difficult breathing.

Script: "There are two ongoing research studies for children with pneumonia and your child may be eligible to participate in one of them. The studies are investigating different treatment regimens for childhood pneumonia, seeing if less antibiotic use is as effective for curing pneumonia. If you are interested in learning more about these studies, I can let the study staff know that it is okay to contact you. If you aren't interested in the studies, that is fine and no one from the study will contact you about them. Your decision to participate in a study will not affect the medical care that your child receives in the hospital. Are you interested in learning more about the studies?"

Prompts: If families ask other questions about the study, including procedures, risks, or benefits, they should be referred to study staff.
